# SCARECROW-LIKE15 interacts with HISTONE DEACETYLASE19 and is essential for repressing the seed maturation programme

**DOI:** 10.1038/ncomms8243

**Published:** 2015-07-01

**Authors:** Ming-Jun Gao, Xiang Li, Jun Huang, Gordon M. Gropp, Branimir Gjetvaj, Donna L. Lindsay, Shu Wei, Cathy Coutu, Zhixiang Chen, Xiao-Chun Wan, Abdelali Hannoufa, Derek J. Lydiate, Margaret Y. Gruber, Z. Jeffrey Chen, Dwayne D. Hegedus

**Affiliations:** 1 Agriculture and Agri-Food Canada, Saskatoon Research Centre, 107 Science Place, Saskatoon, SK, Canada S7N 0X2; 2 Department of Anatomy and Cell Biology, University of Saskatchewan, Saskatoon, SK, Canada S7N 5E5; 3 Key Laboratory of Tea Biochemistry and Biotechnology, Anhui Agricultural University, Hefei, Anhui 230036, China; 4 Department of Botany and Plant Pathology, Purdue University, West Lafayette, IN 47907, USA; 5 Agriculture and Agri-Food Canada, 1391 Sandford Street, London, ON, Canada N5V 4T3; 6 Institute for Cellular and Molecular Biology, Center for Computational Biology and Bioinformatics, University of Texas at Austin, Austin, Texas 78712, USA

## Abstract

Epigenetic regulation of gene expression is critical for controlling embryonic properties during the embryo-to-seedling phase transition. Here we report that a HISTONE DEACETYLASE19 (HDA19)-associated regulator, SCARECROW-LIKE15 (SCL15), is essential for repressing the seed maturation programme in vegetative tissues. *SCL15* is expressed in and GFP-tagged SCL15 predominantly localizes to, the vascular bundles particularly in the phloem companion cells and neighbouring specialized cells. Mutation of *SCL15* leads to a global shift in gene expression in seedlings to a profile resembling late embryogenesis in seeds. In *scl15* seedlings, many genes involved in seed maturation are markedly derepressed with concomitant accumulation of seed 12S globulin; this is correlated with elevated levels of histone acetylation at a subset of seed-specific loci. SCL15 physically interacts with HDA19 and direct targets of HDA19–SCL15 association are identified. These studies reveal that SCL15 acts as an HDA19-associated regulator to repress embryonic traits in seedlings.

## Abstract (web summary)

Epigenetic regulation is critical for controlling gene expression during the transition from embryo to seedling in plants. Here Gao *et al.* report that in *Arabidopsis*, the SCARECROW-LIKE15 protein physically interacts with a histone deacetylase to repress expression of embryonic traits in seedlings.

## Graphical abstract

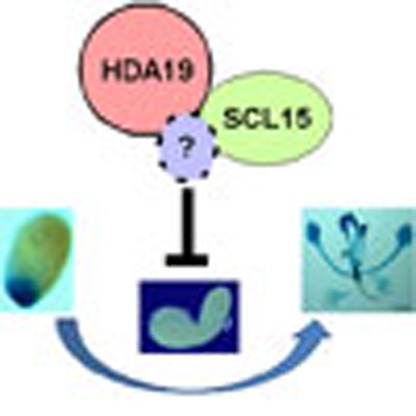

Seed development is an intricate process that begins with double fertilization followed by embryogenesis and seed maturation, the latter being characterized by the expression of embryonic traits such as accumulation of seed storage reserves^1^. The initiation of the seed maturation processes is mainly controlled by three B3 domain transcription factors, namely LEAFY COTYLEDON2 (LEC2), ABSCISIC ACID (ABA) INSENTITIVE3 (ABI3) and FUSCA3 (FUS3). These factors work in consort with the CCAAT-box binding factor (CBF) LEC1, ABA, auxin, gibberellin and sugar signalling^1^,^2^. Transition from the seed maturation to the vegetative phase is thought to be controlled by different mechanisms including environmental cues, hormonal signalling, metabolic changes and transcriptional regulation^3^,^4^,^5^. During seed germination and seedling establishment after release of seed dormancy, the seed maturation programme is repressed so that embryonic traits are not expressed in vegetative tissues.

Chromatin modifications have been implicated in repressing embryonic traits during seed germination and vegetative growth. Inactivation of the bean (*Phaseolus vulgaris*) phaseolin (*phas*) gene in vegetative tissues is attributed to nucleosome positioning over its TATA regions^6^. Histone methylation and acetylation are associated with the chromatin remodelling and activation of the *phas* promoter^7^. The CHD3 chromatin remodelling factor PICKLE (PKL) acts in consort with gibberellic acid to ensure that embryonic traits are not expressed during germination^8^. Furthermore, PKL acts directly upon LEC1, LEC2 and FUS3 that are enriched for trimethylation of histone H3 lysine 27 (H3K27me3)^9^. *Polycomb* (PcG) group proteins play a key role in maintaining the epigenetic states of repressed genes via H3K27me3. The PcG repressive complex 2 (PRC2) is essential for controlling transition from the embryonic phase to the seedling stage by deposition of repressive H3K27me3 mark on seed maturation genes^10^. B3 domain transcription factors and seed maturation genes for 2S albumin, 12S globulin and oleosin possess H3K27me3 marks in vegetative tissues^11^. Derepression of *FUS3* in leaves is directly associated with the PcG protein MEDEA^12^, and repression of *LEC2* during vegetative growth is regulated by a *Repressive LEC2 Element* (*RLE*) that is essential for the deposition of H3K27me3 marks^13^. The VP1/ABI3-LIKE (VAL) B3 proteins VAL1, VAL2 and VAL3 (also known as HSI2, HSL1 and HSL2, respectively)^14^ are essential for the repression of sugar-inducible embryonic traits in seedlings^14^,^15^,^16^. The PHD-L domain of VAL1 was found to repress seed maturation genes through, at least in part, regulation of histone methylation on target genes^17^. Recently, the CW-Zf domain of VAL2 was demonstrated to interact with HISTONE DEACETYLASE19 (HDA19)^18^ and VAL proteins act together with a member of the PRC1, AtBMI1, to initiate the embryo-to-seedling phase transition in *Arabidopsis*^19^. A BRAHMA-containing SNF2 complex in the SWI/SNF family was shown to repress seed genes in *Arabidopsis* leaves^20^. Mutations affecting SDG8 (SET DOMAIN GROUP 8) caused ectopic expression of a subset of seed maturation genes in leaves^21^. Overexpression of miR166 targets, the type III homeodomain-leucine zipper genes *PHABULOSA* (*PHB*) and *PHAVOLUTA* (*PHV*), activated embryonic genes in vegetative tissues^22^. In addition, a trihelix DNA-binding factor ARABIDOPSIS6b-INTERACTING PROTEIN1-LIKE1 (ASIL1) was shown to directly target the GT *cis*-element (5'-GTGATT) of *At2S3 in vitro* and is essential for the repression of the seed regulatory network in *Arabidopsis* seedlings^23^.

Histone acetyltransferases and histone deacetylases (HDACs) play a critical role in the regulation of gene expression and they are commonly associated with other transcriptional regulators to form multi-subunit protein complexes for specific cellular functions^24^. The reversible acetylation and deacetylation of histones are generally accompanied with the activation and silencing of gene expression, respectively^24^. HDA19 was shown to have HDAC activity *in vitro* and to form a multiprotein complex for the repression of gene expression^25^. HDAC activity is thought to be important for the repression of certain seed-specific genes^26^. More specifically, HDA6 and HDA19 contribute redundantly to the repression of embryonic properties after germination^18^,^27^, although the underlying mechanism remains unclear. Here, using a yeast two-hybrid system, we identify SCARECROW (SCR)-LIKE15 (SCL15) as being associated with HDA19. The interaction of SCL15 with HDA19 is confirmed using glutathione *S*-transferase (GST) pull-down, bimolecular fluorescence complementation (BiFC), and co-immunoprecipitation (co-IP) assays. Promoter activity analysis and green fluorescent protein (GFP)-tagged protein localization show that *SCL15* is predominantly expressed in the phloem companion cells (CCs), as well as in specialized cells that are adjacent to the CCs. We demonstrate that SCL15 is required for the repression of a large subset of seed maturation genes and provide evidence that ectopic expression of embryonic genes in *scl15-1* seedlings correlates with the histone H3 hyperacetylation of chromatin at seed-specific loci. Moreover, some of these loci are identified as direct targets of HDA19–SCL15 association. These findings suggest that SCL15 acts as part of an HDA19-associated complex to repress the expression of embryonic genes in seedlings and is involved in the regulation of embryo-to-seedling phase transition.

## Results

### SCL15 is a nuclear GRAS protein

To identify proteins that interact with HDA19, the full-length *Arabidopsis* HDA19 fused to the yeast GAL4 DNA-binding domain (GAL4 DB) was used as bait to screen an *Arabidopsis* seedling cDNA library. A total of 1.6 × 10^6^ yeast transformants were screened yielding nine positive clones. One of these clones encoded SCL15 (At4g36710), which is also designated as AtHAM4 based on its genetic interaction with two *Petunia HAIRY MERISTEM* (HAM) homologues *AtHAM1* and *AtHAM2* (ref. 28). Comparison of deduced amino-acid sequences showed that SCL15 and the HAM homologues AtHAM1, AtHAM2 and AtHAM3 were fairly divergent, showing only 28–31% identity at the amino-acid level (Supplementary Fig. 1). SCL15 shared 86% amino-acid identity with BnSCL1, an auxin responsive HDA19-interacting protein in *Brassica napus*^29^ and a member of the plant-specific GRAS (GIBBERELLIN ACID INSENSITIVE (GAI), REPRESSOR OF GIBBERELLIC ACID INSENSITIVE3 (RGA) and SCR)^30^ protein family. Phylogenetic analysis of all the *Arabidopsis* GRAS proteins confirmed their relationships (Supplementary Fig. 2)^31^. Most GRAS domain proteins identified so far are nuclear proteins and SCL15 was shown to be mainly nuclear localized^31^. To confirm the nuclear localization of SCL15, a transient expression assay was conducted in tobacco epidermal cells with a soluble-modified red-shifted green fluorescent protein (smRS-GFP) translational fusion. The SCL15-smRS-GFP was localized only in the nuclei (Supplementary Fig. 3), indicating that SCL15 is a nuclear-restricted GRAS protein.

### SCL15 physically associates with HDA19

**Figure 1:**
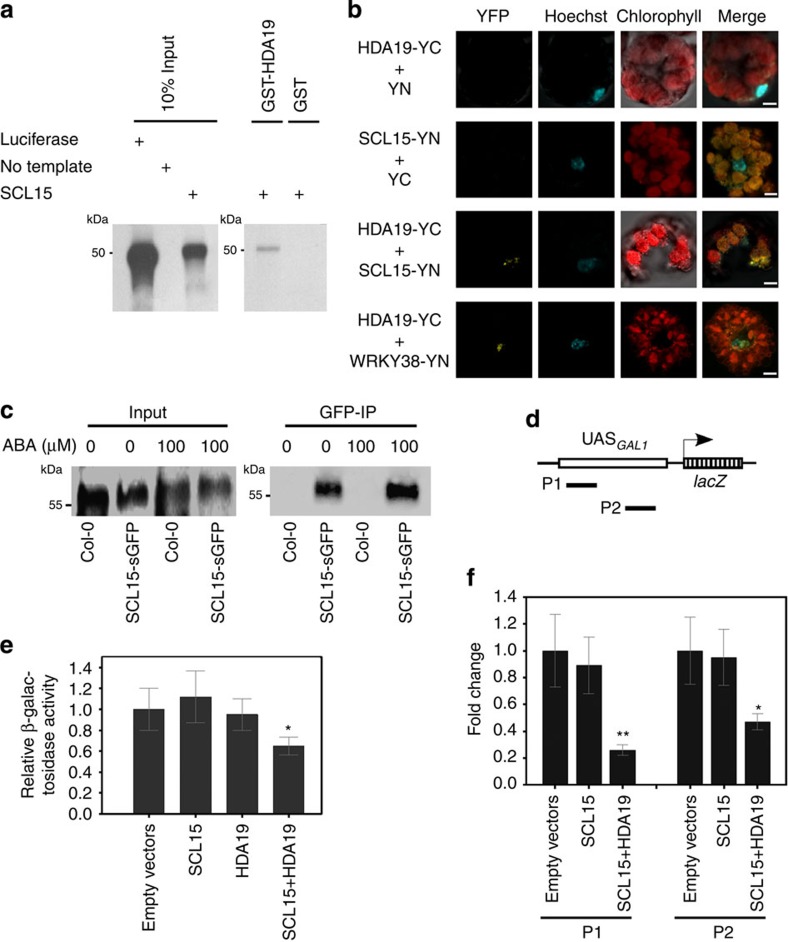
Physical interaction of SCL15 with HDA19. (**a**) GST pull-down assay showing interaction of SCL15 with HDA19 *in vitro*. SCL15 was expressed and radiolabelled using *in vitro* transcription/translation reactions. The translation product (input) was incubated with GST-HDA19 fusion or GST alone. (**b**) BiFC assay displaying SCL15 interaction with HDA19 in the nucleus of *Arabidopsis* protoplasts. Co-expression of SCL15-YN or HDA19-YC with empty vector YC or YN was used as a negative control and co-expression of HDA19-YC and WRKY38-YN served as a positive control for the BiFC assay. Scale bars, 5 μm. (**c**) Co-IP assay in *Arabidopsis* seedlings showing co-precipitation of HDA19 with SCL15-sGFP. Nuclear extracts (Input) from wild-type Col-0 or homozygous transgenic plants stably expressing *SCL15pro::SCL15-sGFP* treated with or without ABA were immunoprecipitated with a polyclonal anti-GFP antibody (GFP-IP) followed by western blot analysis with polyclonal anti-HDA19 antibodies. (**d**) Schematic diagram of the yeast *GAL1pro* containing Gal4-responsive Upstream Activating Sequences UAS_*GAL1*_ and the regions examined after ChIP. (**e**) Co-expression of *HDA19* and *SCL15-GAL4BD* in a *GAL4UAS-GAL1pro::LacZ* yeast reporter line exhibits reduced level of *Lac*Z expression when compared with SCL15-GAL4BD alone or empty vectors. Expression data represent the mean (±s.d.) of three biological replicates (Student's *t*-test, *P<0.05). (**f**) Levels of H3K9ac in the P1 and P2 regions of *GAL1pro*. The fold enrichment of SCL15+HDA19 over SCL15 only is shown and the values are means±s.d. from three biological replicates (Student's *t*-test, *P<0.05, **P<0.01).

To corroborate the physical association between SCL15 and HDA19 detected in the yeast two-hybrid system, three kinds of assays were conducted. First, *in vitro* GST pull-down affinity assays were performed. [^35^S]methionine-labelled SCL15 protein was tested for its ability to interact with recombinant GST–HDA19 fusion protein. SCL15 bound to GST-HDA19 fusion protein, but not to GST alone (Fig. 1a). Second, BiFC assays in *Arabidopsis* protoplasts were carried out. SCL15 was fused to the N-terminal yellow fluorescent protein (YFP) fragment and HDA19 was fused to the C-terminal YFP fragment. A yellow fluorescent signal was observed when SCL15-N-YFP was co-delivered into protoplasts with HDA19-C-YFP (Fig. 1b and Supplementary Fig. 4). Third, a co-IP experiment was performed using nuclear protein extracts from roots of T3 homozygous *SCL15pro::SCL15-sGFP* transgenic plants that were in an *scl15-1* background and had been used for the complementation assays. This assay showed that HDA19 co-precipitated with SCL15-sGFP (Fig. 1c). These results provide good evidence that SCL15 physically interacts with HDA19 in the nuclei of *Arabidopsis* cells.

To test whether SCL15–HDA19 interaction affects the histone acetylation status at a target promoter, a *SCL15-GAL4DB* fusion was used to co-transform a yeast line with *pGAL1-HDA19*. The yeast strain (*GAL4UAS-GAL1pro::lacZ*) harbours an integrated *lac*Z gene under the control of the *GAL1* promoter and is regulated by distinct GAL4-responsive Upstream Activating Sequences (UAS_GAL1_; Fig. 1d). Co-transformation of *SCL15-GAL4DB* with *p426GAL1-HDA19* resulted in reduced β-galactosidase activity when compared with *SCL15-GAL4BD* alone or empty vectors (Fig. 1e). Chromatin immunoprecipitation (ChIP) assays were then conducted to investigate whether co-expression of *SCL15* with *HDA19* led to changes in the histone acetylation state at the *GAL1* promoter. The histone H3K9 acetylation (H3K9ac) levels around the UAS_*GAL1*_ sites were significantly reduced when both *SCL15-GAL4DB* and *p426GAL1-HDA19* were expressed compared with *SCL15-GAL4DB* or the vectors alone (Fig. 1f). This suggests that SCL15 recruits HDA19 to the target promoter via a gene-specific DNA-binding domain to decrease the local histone acetylation levels.

### Phenotypic characterization of *scl15* mutants

**Figure 2:**
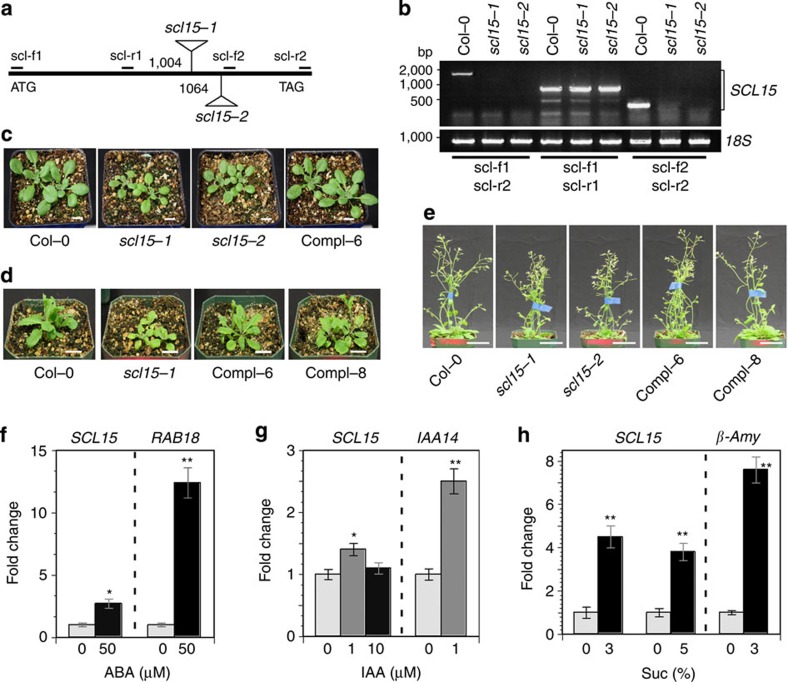
Effects of *SCL15* mutation on plant growth and response to ABA and sugar. (**a**) Schematic representation of the T-DNA insertion alleles of SALK_110871 (*scl15-1)* and GK292A11 (*scl15-2*) in *Arabidopsis*. Numbers indicate positions of T-DNA insertions with respect to the start of translation. The positions of PCR primers for RT–PCR analysis are also indicated. (**b**) RT–PCR analysis of total RNA from leaves of wild-type Col-0 and homozygous *scl15-1* and *scl15-2* plants with several primer pairs. (**c**) Phenotypes of 15-day-old wild-type, homozygous *scl15* mutants and a complemented *SCL15pro::SCL15-GFP* line (Compl-6) plantlets. Scale bar, 1 cm. (**d**) Wild-type, *scl15* mutant and complemented lines (Compl-6 and Compl-8) at 21 days of growth. Scale bar, 2 cm. (**e**) Wild-type, *scl15* mutants and complemented *SCL15pro::SCL15-GFP* lines at 32 days of growth. Scale bar, 5 cm. (**f**) *SCL15* expression 2 days after ABA treatment, (**g**) 1 h after IAA treatment and (**h**) 1 h after sucrose treatment. *RAB18*, *β-Amy* and *IAA14* served as controls to validate the ABA, sugar and auxin responses, respectively. Expression data represent the mean (±s.d.) of three biological replicates (Student's *t*-test, *P<0.05; **P<0.01).

For the functional analysis of *SCL15 in planta*, we obtained two independent T-DNA insertion lines for the *SCL15* gene, namely SALK_110871 (*scl15-1*, also referred to as *Atham4-1* (ref. 28), and GK292A11 (*scl15-2*). Based on the available description of the two T-DNA insertion lines (http://www.arabidopsis.org and http://www.gabi-kat.de), the T-DNA insertion site was located in the coding region of *SCL15* gene, 1,004 and 1,064 bp downstream of the translation start site, respectively (Fig. 2a). Both of the T-DNA insertions were located in the region encoding the PFYRE domain of SCL15 protein. No transcript was detected in the homozygous mutant plants using reverse transcription (RT)–PCR analysis with primers that either spanned the T-DNA insertions or were downstream of the insertion sites. However, RT–PCR products were amplified when using primers located upstream of the T-DNA insertions (Fig. 2b). This result indicates that transcription of the full-length *SCL15* in the mutant plants is disrupted by the T-DNA insertion, but that *scl15-1* and *scl15-2* could be hypomorphic rather than null mutations.

There were no significant abnormalities in plant growth in *scl15-1* and *scl15-2* plants, as previously described^28^,^31^, although they exhibited differences in vegetative growth and development from the wild type before the mid-stage of plant development. The mutant seedlings were smaller than those of the wild type and flowering was delayed by 2 to 3 days in long days when compared with the wild type. The mean rosette leaf numbers of the *scl15* and wild-type plants were 12.8±0.6 and 13.9±0.8 (±indicates standard deviation, *n*=10), respectively (Fig. 2c,d). The *scl15* adult plants at the 32-day-old stage were still shorter (24.9±0.7 cm) than the wild type (30.8±0.8 cm; Student's *t*-test, *P*<0.01; Fig. 2e), but subsequently caught up and had a life cycle from germination to mature seeds of about the same duration as wild-type plants.

To confirm the phenotypes exhibited in *scl15* mutant plants, complementation assays were conducted. The *SCL15* genomic sequence fused to sGFP (*SCL15pro::SCL15-sGFP*) was used to transform *scl15-1* mutant plants. Five independent transgenic lines displaying a 3:1 segregation ratio in T2 populations were selected and two of the T3 homozygous lines, Compl-6 and Compl-8, were chosen for the complementation assays. The SCL15*-*sGFP fusion protein was able to complement the *scl15-1* mutant phenotypes (Fig. 2c–e). There was no difference between the phenotypes of complemented lines and wild-type plants in terms of seedling establishment, flowering and plant growth, indicating that SCL15-sGFP fusion protein is functional.

ABA, auxin, sugar and GA are important cues that guide seed maturation and the transition to seed germination^1^,^3^. The effect of ABA, sugar and auxin on *SCL15* expression was tested. The level of *SCL15* transcript increased substantially in response to ABA and sucrose, whereas *SCL15* expression was only moderately altered by auxin treatment (Fig. 2f–h).

### *SCL15* is predominantly expressed in phloem CCs

**Figure 3:**
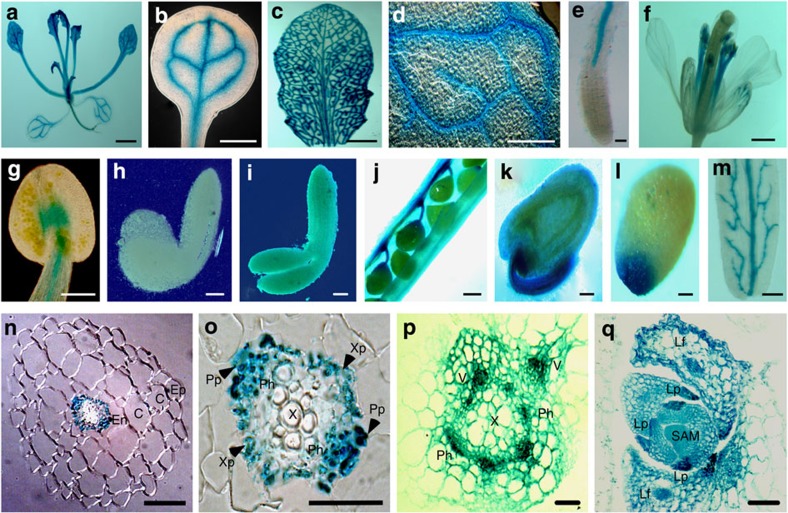
Tissue-specific expression of *SCL15*. (**a**–**e**) GUS activity was assayed in a 28-day-old *SCL15pro::GUS* seedling. (**a**) Whole seedling. (**b**) Cotyledon. (**c**) Fully expanded leaf. (**d**) Portion of leaf surface showing the GUS activity in leaf veins. (**e**) Root tip. (**f**,**g**) GUS staining in floral organs. (**f**) Flower. (**g**) Anther. (**h**–**m**) *pSCL15::GUS* expression in developing seeds. (**h**) Ovules in silique at 4 days post-anthesis (DPA). (**i**) Silique wall at 8 DPA. (**j**) Early bending stage embryo. (**k**) Late bending stage embryo. (**l**) Ovule at 4 DPA. (**m**) Mature seed. (**n**) Transverse section of a root tip showing that GUS activity is exclusively restricted to the vascular bundle. (**o**) Magnified vascular bundle showing that GUS expression is primarily localized at the abaxial side of the vascular bundle. (**p**) Transverse section of a stem. (**q**) Longitudinal section of a shoot apex showing GUS activity in developing leaf primordia and vascular tissues of older leaf primordia. C, cortex; En, endodermis; Ep, epidermis; Lf, leaf; Lp, leaf primordia; Ph, phloem; Pp, phloem-pole pericycle; SAM, shoot apical meristem; V, vascular tissue; X, xylem; Xp, xylem-pole pericycle. Scale bars, 2 nm in **a**–**c**, 200 μm in **d** and **f**, 100 μm in **e**,**g** and **q**, 50 μm in **h**,**i**,**k**,**l** and **n**, 150 μm in **j** and **m**, 20 μm in **o** and **p**.

The specific expression of *SCL15* in vascular tissues, particularly in the root phloem system and chalazal seed coat has been suggested from information in *in silico* databases, the BAR *Arabidopsis* eFP browser (http://bar.utoronto.ca/efp/cgi-bin/efpWeb.cgi) and Genevestigator (https://www.genevestigator.com/gv/plant.jsp; Supplementary Fig. 5). To confirm the expression pattern, we analysed *SCL15* promoter β-glucuronidase (GUS) activity and SCL15-GFP protein localization. The *SCL15* promoter-*GUS* construct was generated by fusing the 2.3-kb fragment directly upstream of the *SCL15* start codon to the *GUS* reporter gene and six independent transformants were analysed. In T1 transgenic *Arabidopsis* plants, strong GUS staining was observed in the vascular tissues of cotyledons, leaves, stamen filaments and primary and lateral roots (Fig. 3a–g). During seed development, *SCL15pro::GUS* expression was not detected in the embryo (Fig. 3h,i), whereas a strong GUS signal was found in the funiculi and chalazal regions of ovules and mature seeds (Fig. 3j–l), as well as the vasculature of silique walls (Fig. 3m).

A more detailed analysis of *SCL15pro::GUS* expression pattern was performed using a dissecting microscope. GUS activity was exclusively restricted to the vascular bundles in mature root and a stronger signal was detected on the abaxial (phloem system) sides of root and stem vascular bundles, whereas no GUS activity was found in the adaxial xylem vessels (Fig. 3n–p). In vegetative apices, GUS activity was observed in the newly formed leaf primordia and the vasculatures of older leaf primordia (Fig. 3q).

**Figure 4:**
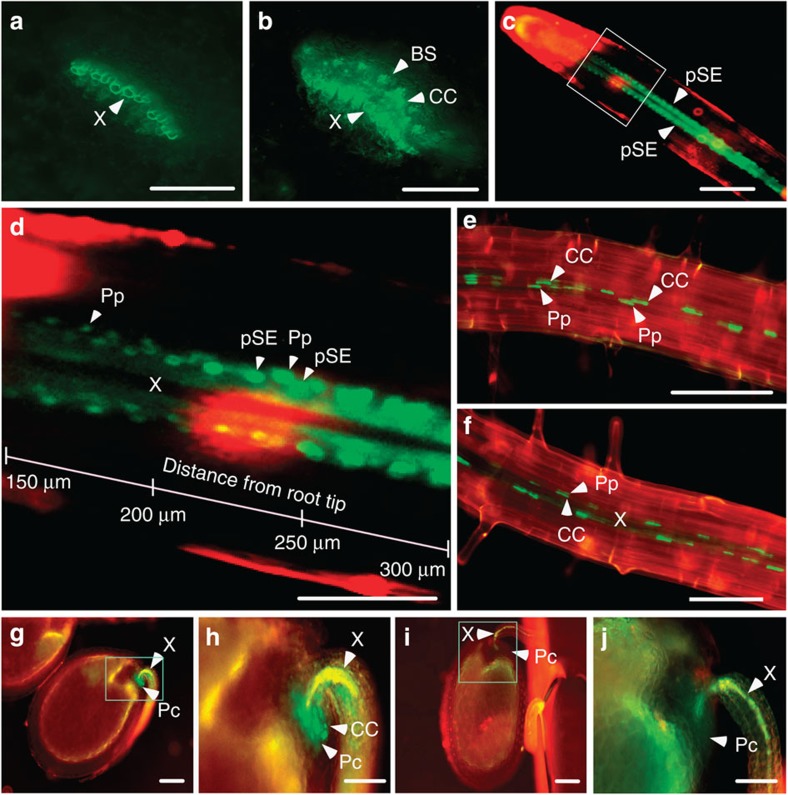
Cell-specific localization of SCL15-sGFP in *Arabidopsis*. (**a**) Leaf petiole cross-sections of wild-type *Arabidopsis*. (**b**) Leaf petiole cross-sections of *SCL15pro::SCL15-sGFP* transgenic plants showing localization of SCL15-GFP fusion in companion cells (CCs) and neighbouring bundle sheath (BS) cells. (**c**) Primary root tip from a 3-day-old *SCL15pro::SCL15-sGFP* seedling displaying GFP localization in protophloem sieve element (pSE). (**d**) The proximal meristem region boxed in **c** is magnified in **d** showing GFP fluorescence is present in pSE as well as in phloem-pole pericycle (Pp) cells. (**e**) SCL15pro::SCL15-sGFP localization in CCs and neighbouring Pp cells of the primary root. One phloem pole is shown when viewed perpendicular to the xylem axis. (**f**) Two phloem poles are shown when viewed perpendicular to the axis of two phloem poles. (**g**) Developing seed from a *SCL15pro::SCL15-GFP* plant showing GFP signal in CCs and neighbouring Pc cells. (**h**) The chalazal seed coat region boxed in **g** is magnified. (**i**) Developing seed from a wild-type Col-0 plant. (**j**) The chalazal seed coat region boxed in **i** is magnified. Pc, placentochalaza; Ps, pigment strand; X, xylem. Scale bars, 100 μm in **a–c**, **e**–**g** and **i**, and 50 μm in **d**,**h** and **j**.

SCL15 localization was examined in vascular tissues of the *scl15-1* mutant expressing *SCL15pro::SCL15-sGFP* that had been used for the complementation assays. The GFP signal occurred in the phloem CCs as well as in the neighbouring tissues. In leaf petiole, the signal was apparent in the CCs and neighbouring bundle sheath (BS) cells (Fig. 4a,b). At the root tip, SCL15-sGFP fluorescence was predominantly localized in protophloem sieve elements and neighbouring phloem-pole pericycle cells (Pp; Fig. 4c,d). In mature roots, the signal was restricted to the nuclei of metaphloem CCs and the Pp cells (Fig. 4e,f). In developing seeds, *SCL15pro::SCL15-sGFP* was expressed in the CCs of chalazal ends of the ovule funiculi, a tissue that connects the ovule to the ovary wall. Interestingly, a strong GFP signal was also present in the placentochalaza, a region that is in close proximity to the CCs at the base of the funiculus vasculature (Fig. 4g–j) and is thought to function as a nutrient unloading zone, particularly for the export of amino acids^32^,^33^.

### SCL15 represses seed maturation genes in vegetative tissue

**Figure 5:**
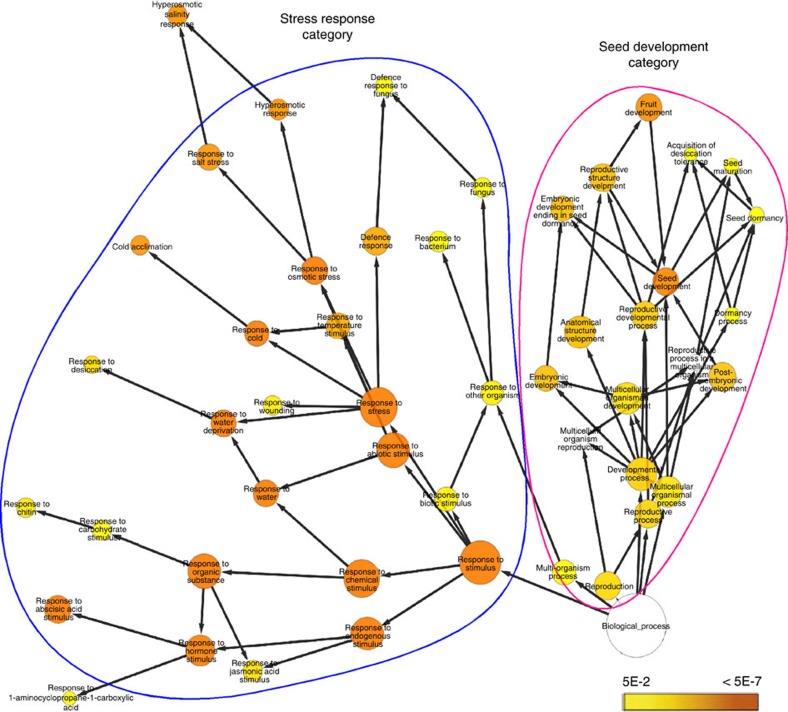
Over-represented gene ontology (GO) categories among genes upregulated in *scl15* mutant seedlings. BiNGO analysis representing over-represented categories of the ontology Biological Process among the genes that are more than threefold (Student's *t*-test (*P*≤0.05) from three biological replicates with a Benjamini-Hochberg false discovery rate of 0.05) upregulated in the *scl15-1* mutant plants compared with the wild type. The two most over-represented GO categories ‘seed development' and ‘stress response' are shown. Coloured nodes ranging from yellow to dark orange represent the levels of significance of the over-represented GO terms with *P* values from 5E-2 to 5E-7 (Hypergeometric test and a Benjamini–Hochberg *P*-value correction for multiple comparisons^34^,^63^).

Microarray analysis on *scl15-1* and wild-type seedlings was performed to obtain global changes in gene expression caused by *SCL15* mutation. Global gene expression patterns in wild-type and the *scl15-1* mutant seedlings were profoundly different, such that 606 genes were at least twofold upregulated and 363 genes were more than twofold downregulated in the mutant relative to the wild type (Student's *t*-test (*P*≤0.05) from three biological replicates with a Benjamini-Hochberg false discovery rate of 0.05; Supplementary Data 1 and 2). To evaluate the upregulated genes, BiNGO analysis was used to assign gene ontology (GO) categories to the 209 genes that showed more than threefold higher expression in the mutant seedlings^34^. Most of the over-represented GO categories in the classification system *biological function* related to reproduction with two distinct subcategories: stress response and seed development (Fig. 5 and Supplementary Fig. 6). The seed development group of genes related to those that are fundamental for various aspects of seed maturation and seed filling, namely genes encoding seed storage proteins (SSPs) and oleosins. The stress response group of genes were linked with environmental stress responses and hormone signalling, such as dehydration, cold stress and ABA signalling. However, a closer analysis of the corresponding genes revealed that most of them are also involved in embryogenesis and seed maturation. Besides reproduction and response to stimuli, a few additional small categories were also over-represented, including genes involved in lipid localization and vascular protein processing. These results suggest that SCL15 represses a large subset of seed maturation genes during the embryonic phase to seedling transition and during seedling establishment.

**Figure 6:**
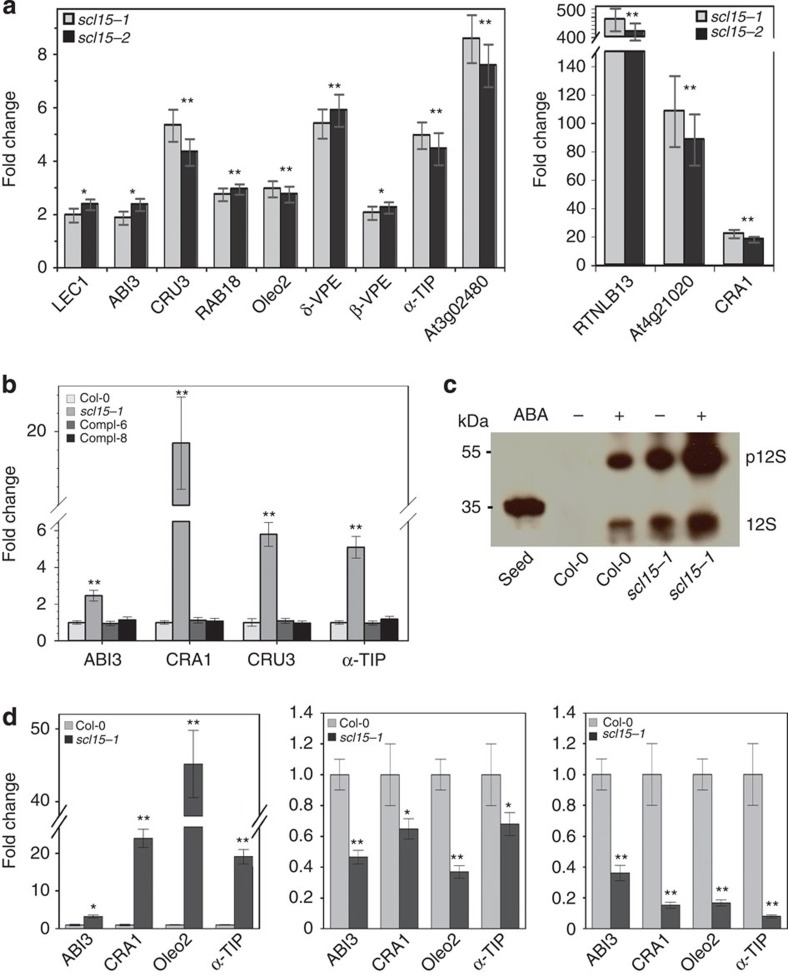
Embryonic genes are ectopically expressed in *scl15* vegetative tissues. (**a**) Ectopic expression of seed maturation genes in *scl15* mutant seedlings. (**b**) Derepression of embryonic genes in *scl15-1* seedlings is restored to the wild-type levels by complementation with *SCL15pro::SCL15-sGFP* (Compl-6 and −8). (**c**) Immunoblot analysis with monoclonal anti-CRA1 antibody showing that accumulation of proglobulin (p12S) and 12S globulin species in *scl15-1* seedlings is increased by treatment with 50 μM ABA for 2 days. (**d**) Expression of seed maturation genes in siliques at the embryo morphogenesis stage (left), the mature (middle) and post-mature stages of embryo development (right). RNA levels are relative to the wild type (onefold). Results represent the mean (±s.d.) of three biological replicates (Student's *t*-test, *P<0.05; **P<0.01).

The DNA microarray results for embryonic genes that were markedly upregulated in *scl15* mutant seedlings were validated using real-time RT–PCR. The tested genes are important in regulating initiation of the seed maturation programme (*LEC1* and *ABI3*), seed reserve trafficking and processing (vacuolar processing enzymes (*VPEs)*, tonoplast intrinsic protein (*α-TIP*) and reticulon family gene (*RTNLB13*)) and accumulation (*CRA1*, *CRU3* and *Oleo2*)^35^. Consistent with the microarray data, all of the genes noted above were upregulated in the mutant seedlings. Several were more than 20-fold upregulated, which included a seed-specific *LEA* (At4g21020) gene, *RTNLB13* and *CRA1* (Fig. 6a, Supplementary Data 1 and Supplementary Table 1). Moreover, the expression of these seed maturation genes in *scl15-1* seedlings expressing *SCL15pro::SCL15-sGFP* did not differ from wild-type plants (Fig. 6b). These results demonstrate that the ectopic expression of a subset of embryonic genes in seedlings is attributable to a defect in *SCL15* expression and suggest that derepression of seed maturation genes in *scl15* seedlings is mediated by upstream regulators in a manner analogous to that occurring in developing wild-type seeds.

To further investigate the extent to which the seed maturation prapram was derepressed in the mutant seedlings, we analysed *scl15-1* for the presence of seed storage 12S globulins. Immunoblot analysis was conducted using monoclonal anti-CRA1 antibody against proteins extracted from 2-week-old seedlings. As shown in Fig. 6c, an unprocessed ∼55-kDa proglobulin (p12S) as well as 12S globulin species accumulated in untreated *scl15-1* seedlings and accumulation of these proteins was increased after treatment with ABA. Globulins were not observed in untreated wild-type seedlings; however, accumulation of these proteins was detected after ABA treatment, although at much lower levels when compared with the ABA-treated *scl15-1* seedlings (Fig. 6c). These findings indicates that in vegetative tissues ABA contributes to the derepression of genes encoding seed storage proteins.

*SCL15* was strongly expressed in the vascular tissues of silique walls, funiculi and chalazal regions of ovules and developing seeds (Figs 3 and 4). Therefore, the expression of various seed maturation genes was examined, including *ABI3*, *α–TIP*, *CRA1* and *Oleo2*, in developing siliques corresponding to embryo morphogenesis (5 days post-anthesis (DPA)), mature (10 DPA) and post-mature (16 DPA) stages of embryo development^32^. Seeds were matured and completely dry after 17 DPA. All seed maturation genes tested in siliques of *scl15-1* mutant at 5 DPA were significantly upregulated, showing similar expression levels to that in seedlings. Heart to linear stage (5 DPA) of seed development is specialized in cellularization and tissue type differentiation before storage reserve accumulation^32^. However, transcripts of these genes were downregulated at the mature stage of embryo development and even further repressed at the post-mature stage compared with the wild type (Fig. 6d).

### HDA19–SCL15 association directly targets embryonic genes

**Figure 7:**
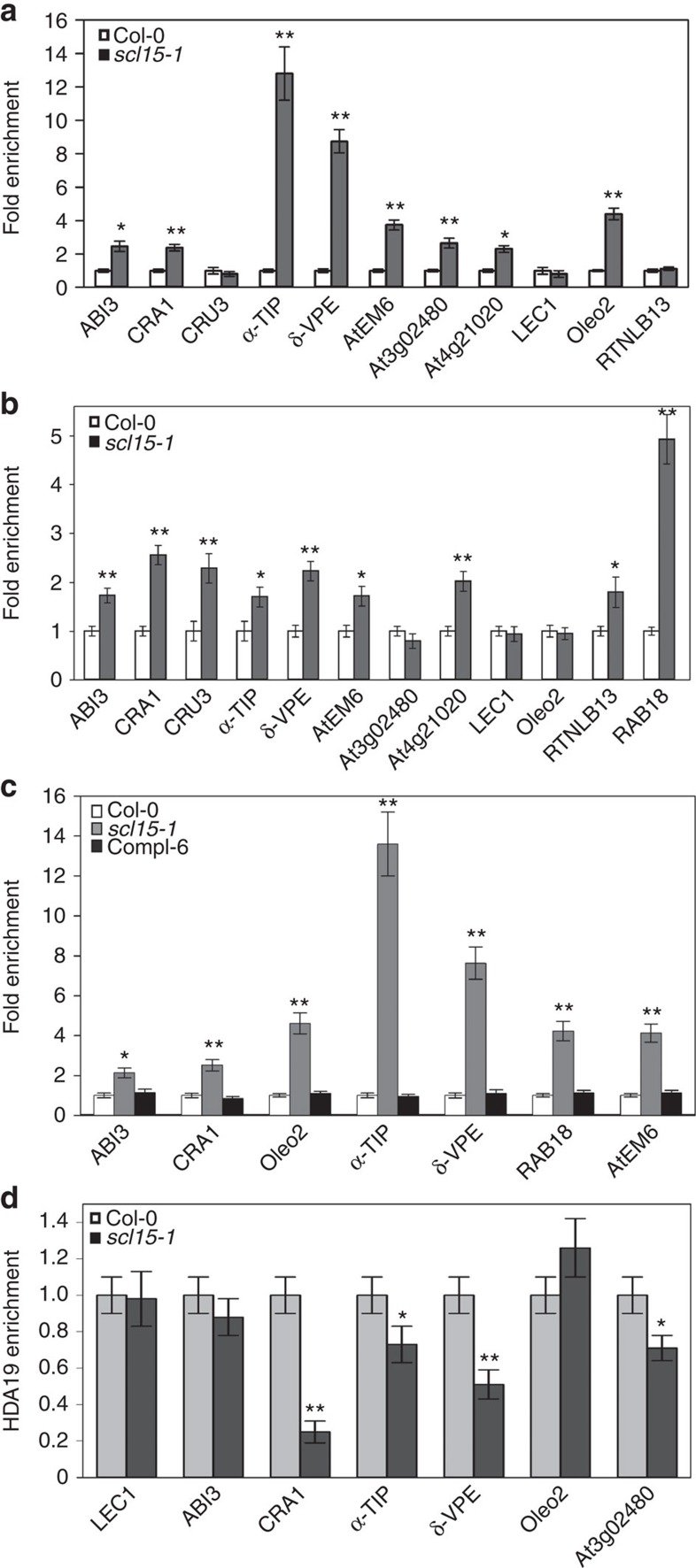
ChIP analysis determines histone acetylation state at seed-specific loci. (**a**) H3K9K14ac levels at the proximal promoter regions and (**b**) the ATG regions of seed maturation genes in wild-type (Col-0) and *scl15-1* mutant seedlings. (**c**) H3K9K14ac levels for seed maturation genes in wild type, the *scl15-1* mutant and a homozygous complemented line stably expressing *SCL15pro::SCL15-sGFP* (Compl-6). (**d**) *In vivo* direct association of HDA19 with seed maturation genes in wild-type and the *scl15-1* mutant seedlings as determined by ChIP-qPCR assays with anti-HDA19 antibodies. The values obtained were normalized with *ACT7* and the relative enrichment of HDA19 binding in wild type relative to the *scl15-1* and/or Compl-6 is shown as the mean±s.d. of three independent experiments (Student's *t*-test, *P<0.05, **P<0.01).

HDA19 is capable of forming a multiprotein complex for the repression of gene expression via histone deacetylation^25^ and is involved in the repression of many seed maturation genes in *Arabidopsis* seedlings by reducing H3K14ac levels^18^. ChIP assay was performed to determine whether *scl15-1* mutation affected histone acetylation at seed-specific loci that were derepressed in mutant seedlings. Increased acetyl H3K9K14 (H3K9K14ac) levels were found at the translational start regions and/or the proximal promoter regions in a subset of seed maturation genes in *scl15-1* seedlings (Fig. 7a,b). These changes in histone acetylation state at the proximal promoter regions for derepressed seed genes in *scl15-1* seedlings were restored to the wild-type levels in the presence of *SCL15pro::SCL15-sGFP* (Compl-6; Fig. 7c). Histone H4 acetylation levels for *ABI3*, *CRA1*, *CRU3*, *AtEM6* and *RAB18* were also elevated in the *scl15-1* mutant (Supplementary Fig. 7); however, H3 and H4 acetylation levels for *LEC1* in the mutant were similar to those in the wild type (Fig. 7a,b). These results indicate that the ectopic expression of many embryonic genes in *scl15-1* mutant seedlings was due, at least in part, to increased histone acetylation levels.

To verify whether HDA19-SCL15 association plays a direct role in the repression of embryonic genes in seedlings, ChIP assays using anti-HDA19 antibodies were performed in wild-type and *scl15-1* seedlings. The *LEC1* gene was used as a negative control because it was derepressed without detectable changes in H3 and H4 acetylation levels in *scl15-1* seedlings. Six embryonic genes encoding SSPs, oleosins and seed reserve trafficking proteins that were markedly derepressed (Fig. 6) and displayed a dramatic increase in histone acetylation in *scl15-1* seedlings (Fig. 7) were examined. HDA19 was significantly enriched in the promoter regions of four genes, including the 12S globulin gene *CRA1*, the protein storage vacuole-localized membrane protein gene *α-TIP*, SSP processing enzyme gene *δ-VPE* and the ABA-responsive seed maturation gene *At3g02480*, in wild type relative to the *scl15-1* mutant seedlings (Fig. 7d), indicating that these genes are direct targets of HDA19–SCL15 interaction. *ABI3* and *Oleo2* did not show significant differences of HDA19 enrichment in the examined promoter regions between wild type and the *scl15-1* mutant.

## Discussion

The important phases of seed development are embryogenesis and seed maturation. The initiation and termination of the seed maturation phase and transition to germination and vegetative growth depend on the maintenance of cell fates and the correct deployment of developmental programmes. Many of the developmental processes active during seed maturation and seed filling are repressed after seed germination and seedling establishment. An increasing body of evidence has shown that chromatin remodelling is the key mechanism underlying repression of embryonic traits in vegetative tissues. The present work demonstrates a novel function for the GRAS family protein SCL15 in preventing the expression of embryonic traits during vegetative growth.

SCL15 possesses conserved domains characteristic of the GRAS family of transcription factors. Localization of SCL15 to the nuclei of plant cells supports its role in transcriptional regulation of gene expression. SCL proteins are a major subfamily of the GRAS proteins, which comprise several groups. Thus far, only a few SCLs have been functionally characterized^31^,^36^,^37^,^38^. Three miR171 targets, namely SCL6/SCL6-IV, SCL22/SCL6-III and SCL27/SCL6-II (ref. 39), but also known as AtHAM3/LOM3, AtHAM2/LOM2 and AtHAM1/LOM1, respectively, were found to play a role in maintaining shoot meristem^28^,^40^. However, SCL6/AtHAM3 was also shown to be involved in the regulation of shoot branching^41^. Recently, SCL27/AtHAM1 was demonstrated to be involved in regulating chlorophyll biosynthesis via direct targeting G(A/G)(A/T)AA(A/T)GT *cis*-elements in the protochlorophyllide oxidoreductase gene^42^. *SCL15* (also referred to as *AtHAM4*) was shown to be functionally redundant with *AtHAM1* and *AtHAM2* based on a secondary meristem phenotype in *Atham1,4; Atham2/+* mutants^28^. Given the fact that *SCL15* is not regulated by miR171, is evolutionarily divergent from *AtHAM1* and *AtHAM2* (ref. 28), and exhibits distinct cell type-specific expression patterns in *Arabidopsis* roots, we suggest that the role of SCL15 in promoting shoot indeterminacy is limited.

Analysis of transcript levels of major embryonic genes and accumulation of SSPs in *scl15* seedlings revealed that SCL15 plays an important role in controlling the embryonic phase to seedling transition through negative regulation of the seed-specific regulatory network. In other studies, several regulators, such as PKL, VALs and ASIL1, contribute to prevent ectopic expression of embryonic genes in seedlings^8^,^14^,^23^. It is noted that these transcriptional regulators may act to selectively repress certain embryonic genes, but not all. The key regulators *LEC1* and *ABI3*, the SSP genes *CRA1* and *CRU3*, and the major oleosin gene *Oleo2* were substantially derepressed in *scl15* seedlings. Transcript levels of δ-*VPE*, *α-TIP* and *RTNLB13*, which are preferentially expressed in developing seeds and are essential for SSP trafficking and processing^35^, were dramatically elevated in *scl15* vegetative tissues. This global gene expression profile in seedlings resembles that in developing seeds at the late phase of embryogenesis. Moreover, the seed storage protein 12S globulin accumulated in *scl15* seedlings. Given the fact that *scl15* mutant plants display no striking abnormalities in development or growth compared with wild type and that embryonic traits were derepressed in the mutant seedlings, we suggest that *Arabidopsis* plants can endure a certain level of seed-specific gene expression in vegetative tissues. Taken together, these results suggest that *SCL15* mutation leads to a metabolic shift in vegetative tissues towards that of late embryogenesis and that SCL15 is required for the negative regulation of seed maturation genes in seedlings.

Expression analysis has shown that regulators for repressing the seed maturation programme, such as PKL, VALs and ASIL1, are not only expressed in vegetative tissues, but also in the embryos of developing seeds^23^. In contrast, *SCL15* is not expressed in the embryo, but is restricted to the vasculature of *Arabidopsis* leaves, roots, stems and siliques. SCL15 was localized in the phloem CCs and neighbouring tissues including leaf BS cells, root Pp cells and the placentochalaza region of the seed coat. This cell type-specific expression patterns leads to the speculation that, in addition to the CCs, the BS cells in leaves and the Pp cells in roots may be important sites for SCL15-regulated ectopic expression of embryonic traits in vegetative tissues. Although there is no direct evidence showing subcellular localization of seed storage reserves in vegetative tissues, the accumulation of vegetative storage proteins has been shown to be restricted to specialized cells including the vascular system in soybean stems, the BS cells in soybean leaves and the bark phloem tissues of trees^43^,^44^.

Most HDACs are found to be part of large multiprotein complexes that are composed of transcriptional repressors and co-repressors^24^. HDAC-associated cofactors appear to be essential for the deacetylation of histones. It has been demonstrated that the specificity of HDAC-associated components impacts substrate selection and ultimately HDAC-mediated repression of target genes^45^. Trichostatin A inhibits HDAC activity during germination and results in elevated expression of embryogenesis-related genes. HDA19 is also known to prevent the expression of embryonic traits after seed germination and has been demonstrated to interact with VAL2 (refs 18, 27); however, little is known about how this regulatory effect is exerted at a molecular level. *In vitro* and *in planta* protein–protein interaction assays indicate that SCL15 acts as a HDA19-associated regulator. Studies on the effect of *scl15* mutation on histone acetylation at seed-specific genes and identification of some embryonic genes as direct targets of HDA19–SCL15 interaction indicate a functional association between SCL15 and HDAC in mediating repression of seed maturation genes in *Arabidopsis* seedlings. Given the contribution of HDA19 to embryonic gene repression after germination, our data suggest that ectopic expression of embryonic traits in *scl15* mutant seedlings was due, at least in part, to increased histone acetylation levels at the seed-specific loci. SCL15 appears to interact with HDA19 to regulate the embryo-to-seedling phase transition. However, HDA6 was also shown to repress embryonic traits in vegetative tissues^27^, and not all of the seed maturation genes that were derepressed in the *scl15* seedlings were identified as direct targets of HDA19–SCL15 interaction. This suggests that other HDACs and/or factors may also contribute to SCL15-associated repression of seed maturation genes.

## Methods

### Plant materials and treatments

*Arabidopsis thaliana* ecotype Col-0 was used as the wild-type plant. T-DNA insertion lines SALK_110871 (*scl15-1)* and GK292A11 (*scl15-2*) (NASC ID: N427947)^46^ were obtained from the ABRC (Ohio State University) and the NASC (University of Nottingham), respectively. *Arabidopsis* plants were grown in pots of soil in a growth chamber that was set to 22 °C day/16 °C night on a 16/8 h day/night cycle and 60% relative humidity. *Arabidopsis* siliques were harvested at the heart to linear (5 DPA), mature (10 DPA) and post-mature (16 DPA) stages of embryo development. Seeds were completely dried after 17 DPA. To grow plants on solid medium, surface-sterilized seeds were kept for 2 days at 4 °C in the dark, placed onto 1/2 MS medium containing 0.7% agar and 1% sucrose, and grown at 22 °C under continuous light. Tobacco (*Nicotiana tabacum*) plants were cultivated in pots of soil in a growth chamber at 22 °C under long day conditions (16/8 h day/night).

For ABA treatments, 14-day-old *Arabidopsis* seedlings were transferred from the 1/2 MS agar-sucrose plates to fresh plates containing the same medium with 0 or 50 μM ABA (A1049; Sigma-Aldrich) and harvested after incubation for 2 days. For the treatment with auxin, GA and sugar, 14-day-old seedlings were incubated in liquid half-strength MS medium supplemented with various concentrations of hormones or sucrose for 1 h. Mock (control) treatment consisted of no or 0.1% ethanol, as the IAA (I2886; Sigma-Aldrich) and GA_3_ (G7645; Sigma-Aldrich) were dissolved in water and 0.1% ethanol, respectively. ChIP analysis was conducted using 3-week-old *Arabidopsis* seedlings grown on solid medium plates as described above. Roots of 14-day-old seedlings that were grown on solid medium and treated with 0 or 100 μM ABA (Sigma-Aldrich) for 4 h were used for co-IP assays. All tissues were harvested and flash frozen in liquid nitrogen and stored at −80 °C until used for RNA isolation and protein extraction.

### Identification of T-DNA insertion lines

Individual plants from T-DNA-tagged lines were genotyped by PCR. The *SCL15*-specific primers scl-f1 and scl-KO-r1 were used for identification of the wild-type allele and T-DNA left border-specific primer LBb1 and the *SCL15*-specific primer scl-f1 were used to detect the mutant allele (Supplementary Data 3 for all primer sequences). The position and structure of the T-DNA insertion site were analysed by PCR, Southern blotting and sequencing of the PCR products that spanned the insertion site. Individual homozygous plants were used for total RNA isolation, RT–PCR and real-time RT–PCR.

### Yeast two-hybrid screening

For construction of the yeast two-hybrid cDNA library, total RNA was isolated from 2-week-old *Arabidopsis* seedlings, converted to cDNA and cloned into a GAL4 activation domain (GAL4 AD) vector pPC86. The SuperScript Plasmid System (Life Technologies) was used for cDNA synthesis and cloning. The open reading frame of the *Arabidopsis HDA19* (Accession no. AY093153; At4g38130) was generated by PCR using primers HDA19-f1 and HDA19-r2, and cloned into the *Sal*I-*Not*I sites of pDBLeu in-frame with the GAL4 DB. This construct served as a bait to screen a cDNA library using the ProQuest Two-Hybrid System (Life Technologies) as previously described^47^. Candidate positive clones were confirmed by yeast retransformation and DNA sequencing analysis. 5′-RLM-RACE (Applied Biosystems**/**Ambion) was undertaken to determine the transcription start sites of candidate genes based on the manufacturer's instructions.

### Alignment and phylogenetic analysis of amino-acid sequences

Alignment of amino-acid sequences was performed using the AlignX programme, Vector NTI Advance 11 suite (Invitrogen), with default settings^48^. A neighbour-joining phylogenetic tree was generated using MEGA 5 (ref. 49) with default parameters.

### *In vitro* protein interaction assays

The coding region of the *Arabidopsis HDA*19 was amplified by PCR using HDA19-f2 and HDA19-r3 primers and inserted in-frame with GST into the *Sal*I and *Not*I sites of vector *pGEX-6P-2* (Amersham Pharmacia). Expression, purification and western blotting analysis of the recombinant HDA19 was carried out as described by Gao *et al.*^50^. The entire coding region of *SCL15* was amplified by PCR using scl-f3 and scl-r3 primers and cloned into the *Hin*dIII and *Xho*I sites of the expression vector *pET-28b* (Novagen) in-frame with the His-Tag sequence. The full-length SCL15 protein labelled with [^35^S]methionine was produced using TNT–Quick Coupled Transcription/Translation System (Promega) according to the manufacturer's instructions. *In vitro* protein interaction was detected using a GST pull-down assays as previously described^50^. Briefly, GST or GST-fusion protein was incubated with [^35^S]Met-labelled translation mixture in a bead-binding buffer (50 mM K-phosphate (pH 7.6), 450 mM KCl, 10 mM MgCl_2_, 10% glycerol, 1% Triton X-100, 1% BSA and 1 μl per 50 ml of buffer of 1:12 diluted protease inhibitors set (Roche Diagnostics)). After incubation for 1 h at room temperature, a 50% slurry of glutathione-Sepharose beads containing 10 mg ml^−1^ of BSA and 4 μg of ethidium bromide was mixed with the interaction mixture followed by gentle rotation for 1 h at 4 °C. After washing six times with bead-binding buffer without BSA or ethidium bromide, but containing the protease inhibitors set, the bound proteins were eluted with 2 × SDS–polyacrylamide gel electrophoresis (PAGE) loading buffer and analysed by 12% SDS–PAGE. After electrophoresis, the gels were dried and subjected to fluorography. Full scans of the fluorographic films are provided in Supplementary Fig. 9.

### BiFC assays

DNA sequences for the N-terminal 173-amino-acid EYFP (N-YFP) and C-terminal 64-amino-acid (C-YFP) fragments were amplified previously by PCR and cloned into the plant expression vectors pOCA30 and pFGC5941 to generate pOCA-N-YFP and pFGC-C-YFP, respectively^51^. N-YFP was also cloned into pFGC5941 to generate a second N-YFP fusion/expression vector (pFGC-N-YFP). *WRKY38*-coding sequence was amplified using primers WRKY38-f1 and WRKY38-r2, digested with *Sac*I/*Spe*I and ligated into the *Sac*I-*Xba*I sites of pOCA-N-YFP to generate an in-frame fusion with N-YFP. The *HDA19*-coding sequence was amplified using primers HDA19-f2 and HDA19-r3, digested with *Spe*I and *Bg*lII and ligated into the *Xba*I-*Bam*HI sites of pFGC-C-YFP to form an in-frame fusion with C-YFP. For generating the SCL15-N-YFP fusion construct, the ∼600 bp 5′ and ∼900 bp 3′ coding sequences of SCL15 were amplified using two pairs of primers scl-f6/scl-r6 and scl-f7/scl-r7, digested with *Sac*I/*Bam*HI and *Bam*HI/*Xba*I, respectively, and sequentially cloned into the same sites of pFGC-N-YFP. *WRKY38* was sub-cloned in the same way as *SCL15* and was used as a positive control for interaction with HDA19 (ref. 51). The plasmids were used for BiFC assays as previously described^52^. Fluorescence and Hoechst 33258 staining were visualized using a Zeiss LSM 710 confocal microscope. In the absence of Hoechst 33258 staining, fluorescence and chlorophyll were excited with YFP excitation and red channel, respectively, on a Zeiss Axio Imager Z1 microscope with ApoTome and AxioCam MRm (Carl Zeiss). The experiments were performed three times with the same results.

### Extraction of nuclear proteins and co-IP assays

Nuclei and nuclear proteins were isolated from roots of 2-week-old Col-0 and *SCL15pro::SCL15-sGFP* (Compl-6) seedlings as previously described^53^. Briefly, root powder was homogenized in cold Buffer A (0.4 M sucrose, 10 mM HEPES, 2.5 mM dithiothreitol, 2 mM EDTA, 1 mM phenylmethylsulfonyl fluoride, 1 protease inhibitor cocktail tablet (Roche Diagnostics) per 50 ml buffer, pH 8). After filtration and centrifugation (14,000*g*) of the extracts, the pellet was resuspended in 1 ml of cold Buffer B (0.25 M sucrose, 10 mM HEPES, 1% Triton X-100, 10 mM MgCl_2_, 1 mM phenylmethylsulfonyl fluoride, protease inhibitor cocktail, pH 8) and centrifuged at 10,000*g* for 10 min at 4 °C. The resultant pellet was resuspended in 300 μl of cold Buffer C (1.7 M sucrose, 10 mM HEPES, 0.15% Triton X-100, 2 mM MgCl_2_, 1 mM phenylmethylsulfonyl fluoride, protease inhibitor cocktail, pH 8). The pellet suspension was then carefully overlaid on top of 500 μl of Buffer C and centrifuged at 16,000*g* for 45 min (4 °C). To release the nuclear proteins from the enriched nuclei, 300 μl of 1 × IP protein extraction buffer (100 mM NaCl, protease inhibitor cocktail, 110 mM KOAc and 0.5% Triton X-100, pH 7.4) was added to the cold nuclei suspension and subsequently sonicated with a sonicator (Qsonica, Model Q55) set at 35% amplitude for five times with 10 s for each sonication. After addition of 2 μl of benzonase (Sigma) to remove nucleic acids, the mixture was centrifuged at 12,000*g* for 30 min at 4 °C and the proteins remaining in the supernatant were quantified.

Co-IP assays were carried out with a kit using Dynabeads (Life Technologies) following the manufacturer's instructions. Briefly, total nuclear proteins (300 μg) were pre-cleared with 1 mg of magnetic beads through continual rotation for 1 h at 4 °C. Polyclonal anti-GFP antibody (7.5 μg; Clontech, Catalogue No. 632592) was coupled to 1.5 mg of magnetic beads at a dilution of 1:200 following the manufacturer's instructions and then incubated with the pre-cleared nuclear protein extract by rotating for 30 min at 4 °C. The immobilized proteins on the GFP-coupled beads were washed four times and subsequently eluted for immunoblotting using anti-HDA19 antibodies^54^ at a dilution of 1:2,000 and HRP-conjugated goat anti-rabbit IgG (Invitrogen, Catalogue No. 65–6120) as the secondary antibody at 1:30,000 dilution. The Clarity Western ECL Substrate system (Bio-Rad) was used for immunodetection of HDA19. Full scans of the western blots are shown in Supplementary Fig. 10.

### ChIP assays in yeast

The effector plasmid *SCL15-GAL4BD* was constructed by ligating the amplified *SCL15* product (primers scl-f4 and scl-r4) between the *Sal*I and *Not*I sites of the vector *pDBLeu* (Life Technologies) in-frame with the *GAL4BD* sequence. To make the construct *p426GAL1-HAD19*, plasmid *pDBLeu-HDA19* was digested with *Sal*I and *Not*I and the *HDA19* fragment cloned between the *Sal*I and *Not*I sites of the vector *p426 GAL1* (Life Technologies). MaV203 yeast cells, expressing an integrated *lac*Z reporter gene driven by the *GAL1* promoter (*GAL1pro*; Life Technologies), were transformed with either the effector *SCL15-GAL4BD* only, both *SCL15-GAL4BD* and *p426GAL1-HDA19*, or empty vectors *pDBLeu* and *p426 GAL1*. The β-galactosidase activity was measured using chlorophenol red-β-D-galactopyranoside. Quantitative data (mean±s.d.) were determined from three independent assays. The *GAL1pro* contains distant GAL4BD sites located in the 365-bp upstream activation sequence UAS_G_ designated as UAS_*GAL1*_ (ref. 55). ChIP experiments were performed as previously reported^47^ using anti-acetyl-histone H3K9 antibody (07–352; Millipore). DNA enrichment was analysed by real-time quantitative PCR (qPCR) using specific primers. Three replicates of the ChIP assay were carried out on different preparations of nuclei.

### ChIP assays in *Arabidopsis*

ChIP experiment was conducted as described previously^56^. Chromatin extract was prepared from seedlings treated with formaldehyde. After being sheared to an average length of 500 bp by sonication, the chromatin was immunoprecipitated with specific antibodies, including anti-acetyl-histone H3K9K14 (06–599; Millipore), anti-acetyl-histone H4K5K8K12K16 (06–866; Millipore) and anti-HDA19 (ref. 54). qPCR was carried out to detect the DNA cross-linked to the immunoprecipitated proteins. Primers used for qPCR analysis in ChIP assays were designed within 350 bp around the transcription initiation sites (promoter region) or to flank the ATG translation start site (ATG region; Supplementary Fig. 8). Primer sequences are given in Supplementary Data 3. *ACT7* was used as internal control for normalization^7^. Each immunoprecipitation was replicated a minimum of three times and each sample was quantified in triplicate. All results were shown as means±s.d. of at least three biological replicates.

### Plasmid construction

For subcellular protein localization, the construct *35Spro::smRS-GFP* was generated in the binary vector *pCAMBIA*^57^ by subcloning the cDNA (U70496) that encodes the smRS-GFP^58^. The *SCL15* open reading frame was amplified by PCR and subcloned into the *EcoR*I-*Sac*II sites of the plasmid *35Spro::smRS-GFP* in-frame with the N-terminus of smRS-GFP generating *35Spro::SCL15-smRS-GFP*. Plasmid *35Spro::smRS-GFP* was used as the negative control for transient expression in *Nicotiana tabacum* epidermal cells^59^.

For promoter expression studies, 2,353 bp of the *SCL15* promoter including 299 bp of 5′ untranslated region was amplified using primer pairs scl-f8 and scl-r8 and cloned into the binary vector *pBI121* by replacing the *CaMV 35S* promoter. The *SCL15pro::GUS* plasmid was introduced into *Agrobacterium tumefaciens* strain GV3101 followed by transformation into *Arabidopsis* plants using the floral dipping method^60^.

For complementation of the *scl15* mutant, the *SCL15* gene sequence (including 2,051 bp of promoter, the 5′ untranslated region and 1,458 bp of coding region without the TAG stop codon) was synthesized in frame with 717 bp of *sGFP* by GeneArt Gene Synthesis (Life Technologies, Canada), in which a ten-residue flexible polypeptide linker (GGGGSGGGGS) was inserted before *sGFP*, and cloned into a modified *pZP121* binary vector^57^. The resulting *SCL15pro::SCL15-sGFP* construct was used to transform homozygous *scl15-1* mutant plants. Multiple independent transgenic lines that displayed a 3:1 segregation ratio in T2 populations were selected. The T3 homozygous lines Compl-6 and Compl-8 were chosen for the complementation assays.

### Histochemical and microscopic analysis

For subcellular localization of the SCL15:smRS-GFP fusion, transiently transformed *N. tabacum* leaves were incubated with 50 μg ml^−1^ 4',6-diamidino-2-phenylindole (DAPI; Life Technologies) for nuclei staining. Leaf tissues were stained with DAPI for 30 min. GFP signal was observed on a Zeiss Axio Imager Z1 microscope with ApoTome and AxioCam MRm and AxioVision Rel. 4.7 Imaging System (Carl Zeiss) with excitation of 490 and 383 nm for GFP and DAPI, respectively. The images shown are representative of at least three independent experiments.

For *SCL15pro::GUS* activity assay, whole *Arabidopsis* seedlings or individual organs were incubated with GUS staining solution (10 mM phosphate buffer, 10 mM EDTA, 0.1% (v/v) Triton X-100, 1 mM potassium ferricyanide, 1 mM potassium ferrocyanide, 1 mg per 1 ml of X-Gluc (5-bromo-4-chloro-3-indolyl-β-D-glucuronide) from 50 mg ml^−1^ stock in dimethylformamide). Samples were mounted in water under a coverslip and viewed using a Zeiss Axio Imager Z1 microscope. For histological analysis of transverse sections of *Arabidopsis* tissues, GUS staining was performed as described by Sieburth and Meyerowitz^61^. Images were captured using an Axioplan Universal microscope with a Digital CCD camera and AxioCam ICc 1 and AxioVision Release 4.7 software.

For anatomical analysis of *SCL15pro::SCL15-sGFP* plants, whole seedlings were stained briefly with 10 μg ml^−1^ propidium iodide (Sigma-Aldrich), mounted in water under glass coverslips, and visualized on a Zeiss Axio Imager Z1 microscope with ApoTome and AxioCam MRm software. The excitation wavelengths for GFP and propidium iodide were 561 nm and 488 nm, respectively.

### Microarray and data analysis

Wild-type Col-0 and *scl15-1* seeds were sterilized and sown onto 1/2 MS agar-sucrose plates. Total RNA was isolated from 14-day-old seedlings using the RNeasy Plant mini kit (Qiagen). An *Arabidopsis* 70-mer oligo-gene array containing 26,090 annotated genes was used for microarray analysis. The GeneChip arrays were developed by Qiagen/Operon and printed on Super Amine slides (Telechem International) at the Microarray and Proteomics Facility, University of Alberta, Edmonton, AB, Canada. Total RNA was amplified and probe was labelled using an Ambion Amino Allyl MessageAmp II RNA amplification kit (Ambion) according to the manufacturer's instructions as described previously^62^. Total RNA from three independent biological replicates were used. Briefly, fluorescent cDNA probes were synthesized from RNA and labelled with Cy3- or Cy5-dCTP using CyDye Post-Labelling Reactive Dye Packs (GE Healthcare). Hybridization and washing were performed according to the Corning Epoxide Coated Slides instruction manual (Corning Life Sciences). The labelled antisense RNA was fragmented in Ambion's fragmentation buffer (Applied Biosystems). After addition of 45 μl of DIG Easy Hybridization solution (Roche, Diagnostics) and 3 μl of blocking buffer (GE Healthcare) followed by incubation for 2 min at 65 °C, samples were hybridized for 16–18 h at 37 °C using a MAUI hybridization system (BioMicro Systems). Microarray images were analysed using Array-Pro Analyzer software (Media Cybernetics). The data were then extracted, log transformed, quantile normalized and corrected for background using Robust Multiarray Analysis. Statistical analysis of gene expression data was based on the Student's *t*-test (*P*≤0.05) and a Benjamini-Hochberg false discovery rate multiple testing correction with cutoff of 0.05 (ref. 63). Differentially expressed genes were considered to be those exhibiting at least a twofold change. GO categories that were significantly over-represented among upregulated genes in the *scl15-1* seedlings were determined using the Biological Network Gene Ontology tool 2.4 (BiNGO 2.4) plugin^34^ for Cytoscape 2.8 (ref. 64) with default settings (hypergeometric test and a Benjamini-Hochberg multiple testing correction^63^).

### qRT–PCR analysis

Total RNA was extracted from *Arabidopsis* seedlings or siliques using the RNeasy Plant Mini Kit (Qiagen) and genomic DNA was eliminated from RNA samples with DNase I (amplilification grade; Life Technologies). Total RNA was reversed transcribed using the SuperScript III First-Strand Synthesis SuperMix for qRT–PCR (Life Technologies) following the manufacturer's instructions. Real-time RT–PCR was performed using the Platinum SYBR Green qPCR SuperMix-UDG (Life Technologies) on an ABI Prism StepOnePlus Real-time PCR System (Applied Biosystems) or using the SsoFast EvaGreen Supermix (Bio-Rad) in a CFX96 Touch Real-Time PCR Detection System (Bio-Rad) according to the manufacturer's instructions. *ACT2* and *Ef-1α* were used as the reference genes. Each pair of primers produced a single amplicon of the expected size and melting temperature. The gene-specific primers for *LEC1*, *ABI3*, *CRU3*, *RAB18*, *Oleo2*, *Ef-1α* and *ACT2* have been described previously^23^ and those for other genes are listed in Supplementary Data 3. Data were analysed using the StepOne Software v2.0 (Applied Biosystems) or the CFX Manager 3.1 (Bio-Rad). All samples were run with triplicate and results present are the mean (±s.d.) of three biological replicates.

### Immunoblotting

*Arabidopsis* 12S globulin was purified by HPLC and a monoclonal anti-12S globulin CRA1 was generated by the Animal Care Unit, Western College of Veterinary Medicine, University of Saskatchewan, Saskatoon, Canada. 12S globulin were purified from leaves of 2-week-old Col-0 and *scl15-1* plants following treatment with or without 50 μM ABA for 2 days by employing a RuBisCO depletion and antibody-capture technique. Briefly, 100 μg of ground leaf tissues was combined with 500 μl protein extraction buffer (7 M urea, 2 M thiourea, 50 mM Tris-HCl, pH 9.0, 0.2% Triton X-100, one Complete Protease Inhibitor Cocktail Tablet (Roche Diagnostics) per 30 ml). Following homogenization with a micropestle and centrifugation at 7,800*g* at 4 °C for 20 min, supernatants were clarified by passage through a 0.45-μm syringe filter and centrifugation at 10,000*g*, 4 °C for 1 min. For each supernatant, 500 μg protein was subjected to Rubisco depletion using Seppro Rubisco Spin Columns (Sigma-Aldrich) and then used directly for antibody capture using rabbit anti-globulin polyclonal-coupled Dynabeads Protein G (Life Technologies) according to the manufacturer's instructions. Enriched 12S globulin species were eluted, boiled and resolved by 15% SDS–PAGE for silver staining and immunoblotting analysis. For detection of globulin species, a 1:1,000 dilution of monoclonal anti-CRA1 antibody and a 1:5,000 dilution of anti-mouse-HRP (Bio-Rad,Catalogue #170–6516) were employed along with Immobilon Western Chemiluminescence HRP Substrate (Millipore) according to the manufacturer's instructions. A full scan of the immunoblot is presented in Supplementary Fig. 11.

## Additional information

**Accession codes:** The microarray data were deposited to NCBI Gene Expression Omnibus under the accession code GSE67395.

**How to cite this article:** Gao, M.-J. *et al.* SCARECROW-LIKE15 interacts with HISTONE DEACETYLASE19 and is essential for repressing the seed maturation programme. *Nat. Commun.* 6:7243 doi: 10.1038/ncomms8243 (2015).

## Supplementary Material

Supplementary Figures and Table Supplementary Figures 1-11 and Supplementary Table 1

Supplementary Data 1 Genes up-regulated in the scl15-1 mutant seedlings

Supplementary Data 2 Genes down-regulated in the scl15-1 mutant seedlings

Supplementary Data 3 Sequences of the oligonucleotides used in this study
