# Supplementary material for: SCARECROW-LIKE15 interacts with HISTONE DEACETYLASE19 and is essential for repressing the seed maturation programme
Source: Nat Commun. 2015 Jul 1;6:7243. doi: 10.1038/ncomms8243 (PMC4507008; doi:10.1038/ncomms8243)
Supplement: Supplementary Figures and Table — Supplementary Figures 1-11 and Supplementary Table 1 [file ncomms8243-s1.pdf]

```
SCL15    (1) M-----------------------------------------------------------K
BnSCL1   (1) M-----------------------------------------------------------K
SCL27    (1) MPLSFERFQGEGVFGLSSSSFYSDSQKIWSNQDKTEAKQEDLGYVVG-GFLPEPTSVLDAL
SCL22    (1) MPLPFEQFQGKGVLGFLDSSSSPGYKIWANPEKLHGRVEEDLCFVVNNGGFSEPTSVLDSV
SCL6     (1) MPLPFEEFQGKGISCFSSF-SSSFPQPPSSPLLSHRKARGGEEEEEE-VPAAEPTSVLDSL

SCL15    (3) IPASSPQDTTNNNNNTNSTDSNH------------LSMDEHVMRSMDWDSIMKELELD---
BnSCL1   (3) LQASSPQDNQPSNTTNNSTDSNH------------LSMDEHAMRSMDWDSIMKELEVD---
SCL27   (61) RSPSPLASYSSTTTTLSSSHGGGGTTVTNTTVTAGDDNNNNKCSQMGLDDLDGVLSASSPG
SCL22   (62) RSPSPFVSSSTTTLSSSHGGPSGGG-----AAAATFSGADGKCDQMGFEDLDGVLSGGSPG
SCL6    (60) ISPTSSSTVSSSHGGNSAVGGGG------------DATTDEQCGAIGLGDWEEQVPHD---

SCL15   (49) DDSAPNSLKTGF------------------------------------TTTTTDSTI---L
BnSCL1  (49) DDSAPYQLQPS-------------------------------------------S------
SCL27  (122) QEQSILRLIMDPGSAFGVFDPGFGFGSGSGPVSAPVSDNSNLLCNFPFQEITNPAEA---L
SCL22  (118) QEQSIFRLIMAGDVVDPGSE---FVGFDIGSGSDPVIDNPNPLFGYGFPFQNAPEEEKFQI
SCL6   (106) HEQSILGLIMG--------------------------------------DSTDPS-----L

SCL15   (71) PLYA---VDSNLPGFPDQIQPSD-------FESSSDVYPGQNQTTGYGFNS----------
BnSCL1  (61) ---------FNLPVFPD--------------IDSSDVYPGPNQITGYGFNS----------
SCL27  (180) INPSNHCLFYNPPLSPPAKRFNSG-SLHQPVFPLSDPDPGHDPVRRQHQFQFPFYHNNQQQ
SCL22  (176) SINPNPGFFSDPPSSPPAKRLNSG-------------QPGSQHLQWVFPFSDPGHESHDP-
SCL6   (124) ELNS---ILQTSPTFHDSDYSSPGFGVVDTGFGLDHHSVPPSHVSGLLINQSQTHYTQNP-

SCL15  (112) ------------------LDSVDNGGFDFIEDLIRVVDCVESDEL------QLAQVVLSRL
BnSCL1  (89) ------------------LDSVDNGGFDYIEDLIRVVDCIESDEL------HLAHVVLSQL
SCL27  (240) QFPSSSSSTAVAMVPVPSPGMAGDDQSVIIEQLFNAAELIGTTGNNNGDHTVLAQGILARL
SCL22  (223) -------FLTPPKIAG-EDQNDQDQSAVIIDQLFSAAAELTTNGGDN--NPVLAQGILARL
SCL6   (181) ----AAIFYGHHHHTPPPAKRLNPGPVGITEQLVKAAEVIESDT-------CLAQGILARL

SCL15  (149) NQRLRSPA-------GRPLQRAAFYFKEALGSFLTGSNRNPIRLSSWSEIVQRIRAIKEYS
BnSCL1 (126) NQRLQTSA-------GRPLQRAAFYFKEALGSLLTGTNRN--QLFSWSDIVQKIRAIKEFS
SCL27  (301) NHHLNTSSNH-----KSPFQRAASHIAEALLSLIHNESSPP--LITPENLILRIAAYRSFS
SCL22  (274) NHNLNNNNDDTNNNPKPPFHRAASYITEALHSLLQDSSLSPPSLSPPQNLIFRIAAYRAFS
SCL6   (231) NQQLSSPV-------GKPLERAAFYFKEALNNLLHNVSQT----LNPYSLIFKIAAYKSFS

SCL15  (203) GISPIPLFSHFTANQAILDSLSSQSSSPFVHVVDFEIGFGGQYASLMREITEKSVSGGF--
BnSCL1 (178) GISPIPLFSHFTANQAILDSLSSQSSSPFVHVVDFEIGFGGQYASLMREIAEKSANGGF--
SCL27  (355) ETSPFLQFVNFTANQSILESCNESGFDR-IHIIDFDVGYGGQWSSLMQELASGVGGRRRNR
SCL22  (335) ETSPFLQFVNFTANQTILESFEGFDR---IHIVDFDIGYGGQWASLIQELAGKRNRSSS--
SCL6   (281) EISPVLQFANFTSNQALLESFHGFHR---LHIIDFDIGYGGQWASLMQELVLRDNAAPLS-

SCL15  (262) -----LRVTAVV--AEECAVETRLVKENLTQFAAEMKIRFQIEFVLMKTFEMLSFKAI---
BnSCL1 (237) -----LRVTAVV--AEDCAVETRLVKENLTQFAAEMKIRFQIEFVLMKTFEILSFKAI---
SCL27  (415) ASSLKLTVFAPPPSTVSDEFELRFTEENLKTFAGEVKIPFEIELLSVELLLNPAYWPL-SL
SCL22  (391) APSLKITAFASP-STVSDEFELRFTEENLRSFAGETGVSFEIELLNMEILLNPTYWPLSLF
SCL6   (338) ---LKITVFASP--ANHDQLELGFTQDNLKHFASEINISLDIQVLSLDLLGSISWP-----

SCL15  (313) RFVEGERTVVLI--SPAIFRRLSGITDFVNNLRRVSPKVVVFVDSEGWTEIAGSGSFRREF
BnSCL1 (288) RFVDGERTVVLI--SPAIFRRVIGIAEFVNNLGRVSPNVVVFVDSEGCTETAGSGSFRREF
SCL27  (475) RSSEKEAIAVNLPVNSVASGYLPLILRFLKQLS----PNIVVCSDRGCDRN--DAPFPNAV
SCL22  (451) RSSEKEAIAVNLPISSMVSGYLPLILRFLKQIS----PNVVVCSDRSCDRN-NDAPFPNGV
SCL6   (389) NSSEKEAVAVNI--SAASFSHLPLVLRFVKHLS----PTIIVCSDRGCERT--DLPFSQQL
```

```
                                        *******************************
   SCL15  (372) VSALEFYTMVLESLDAAAPPGDLVKKIVEAFVLRPKISAAVETAADRRHTGEMTWREAFCA
  BnSCL1  (347) VSAFEFYTMVLESLDAAAPPGDLVKKIVETFLLRPKISAAVETAANRRSAGQMTWREMLCA
   SCL27  (530) IHSLQYHTSLLESLDANQNQDDSS---IERFWVQPSIEKLLMKRHRWIERSP-PWRILFTQ
   SCL22  (507) INALQYYTSLLESLDSGNLNNAEAATSIERFCVQPSIQKLLTNRYRWMERSP-PWRSLFGQ
    SCL6  (442) AHSLHSHTALFESLDAVNANLDAMQK-IERFLIQPEIEKLVLDRSRPIERPMMTWQAMFLQ
                ° ° ° ° ° ° ° ° ° ° ° ° °                         • • • • • • • • • • •

   SCL15  (433) AGMRPIQQSQFADFQAECLLEKAQVRGFHVAKRQGE---LVLCWHGRALVATSAWRF---
  BnSCL1  (408) AGMRPVQLSQFADFQAECLLEKAQVRGFHVAKRQGE---LVLCWHGRALVATSAWRF---
   SCL27  (587) CGFSPASLSQMAEAQAECLLQRNPVRGFHVEKRQSS---LVMCWQRKELVTVSAWKC---
   SCL22  (567) CGFTPVTLSQTAETQAEYLLQRNPMRGFHLEKRQSSSPSLVLCWQRKELVTVSAWKC---
    SCL6  (502) MGFSPVTHSNFTESQAECLVQRTPVRGFHVEKKHNS---LLLCWQRTELVGVSAWRCRSS
                ••••••••••••••••••••••••••••••••••••••••••••••••••••••••••••
```

Supplementary Figure 1. Comparison of the deduced amino acid sequences of SCL15 with those of HAIRY MERISTEM (HAM) homologs SCL27 (ATHAM1), SCL22 (ATHAM2) and SCL6 (ATHAM3) and *Brassica napus* BnSCL1. Identical amino acids are shown as red letters on a yellow background. Conserved amino acids are highlighted in blue. Amino acids with weak similarity are indicated in green. The two leucine heptad repeats (LHRI and LHRII), VHIID motif, PFYRE and SAW motif are underlined with rectangles, double lines, circles, and dots, respectively. Upside down triangles indicate residues that define the leucine heptad repeats. A potential nuclear localization signal is indicated by asterisks. Accession numbers for the sequences are as follows: SCL15 (At4g36710), BnSCL1 (AY66405), SCL27 (At2g45160), SCL22 (At3g60630), and SCL6 (At4g00150).

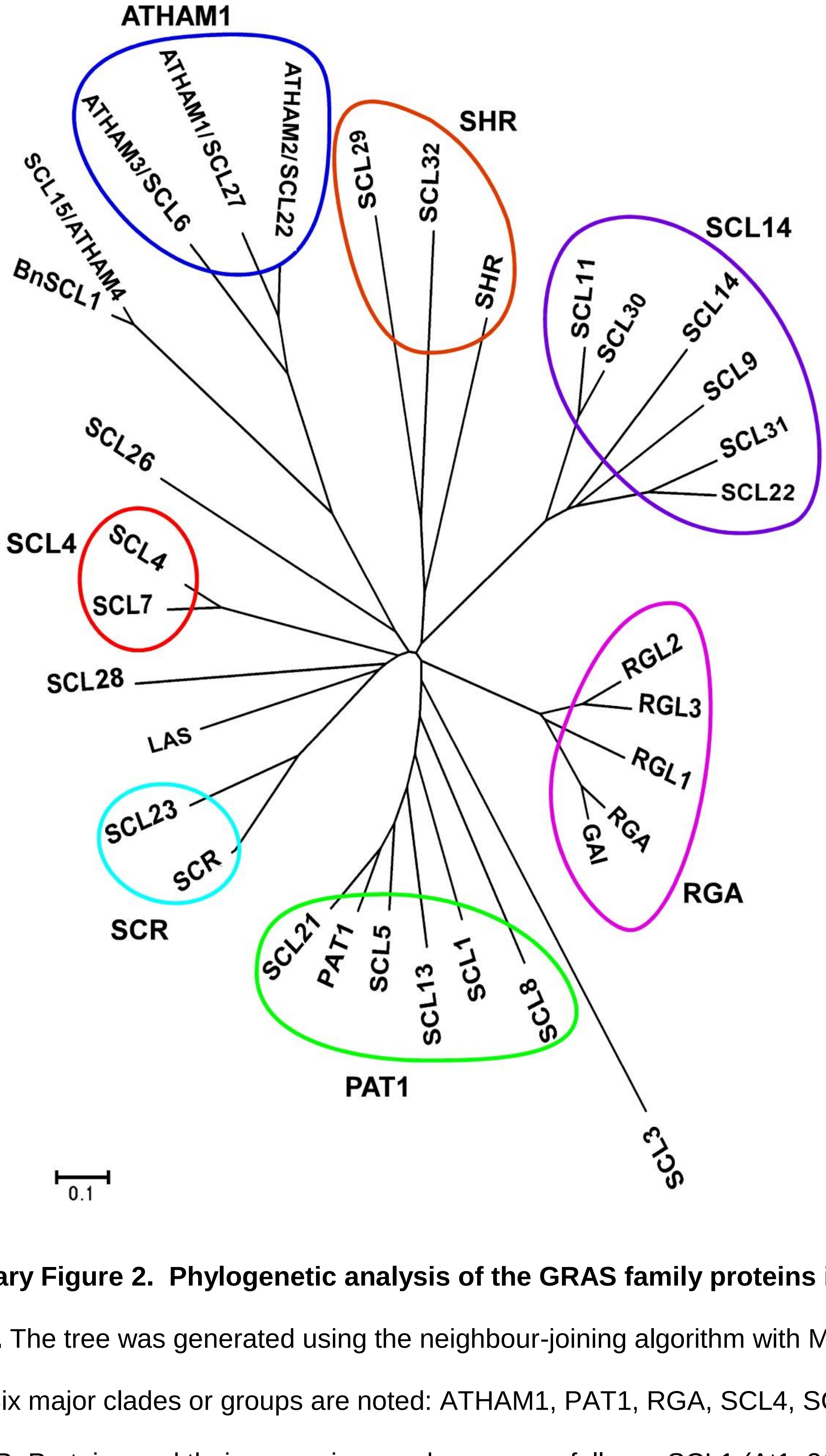

Supplementary Figure 2. Phylogenetic analysis of the GRAS family proteins in *Arabidopsis*. The tree was generated using the neighbour-joining algorithm with MEGA version 5[46]. Six major clades or groups are noted: ATHAM1, PAT1, RGA, SCL4, SCL14, SCR and SHR. Proteins and their accession numbers are as follows: SCL1 (At1g21450), SCL3 (At1g50420), SCL4 (At5g66770), SCL5 (At1g50600), SCL6 (also designated as LOM3 or AtHAM3 (At4g00150), SCL7 (At3g50650), SCL8 (At5g52510), SCL9

(At2g37650), SCL11 (At5g59450), SCL13 (At4g17230), SCL14 (At1g07530), SCL15/ATHAM4 (At4g36710), SCL21 (At2g04890), SCL22/AtHAM2) (At3g60630), SCL23 (At5g41920), SCL26 (At4g08250), SCL27 (also designated as LOM1 or AtHAM1) (At2g45160), SCL28 (At1g63100), SCL29 (At3g13840), SCL30 (At3g46600), SCL31 (At1g07520), SCL32 (At3g49950), SCL33 (At2g29060), GAI/RGA2 (At1g14920), LAS (At1g55580), PAT1 (At5g48150), RGA/RGA1 (At2g01570), RGL1 (At1g66350), RGL2 (At3g03450), RGL3 (At5g17490), SCR (At3g54220), SHR (At4g37650). *Brassica napus* BnSCL1 (AY66405) is also included for comparison. Scale bar represents 0.1 amino acid substitutions.

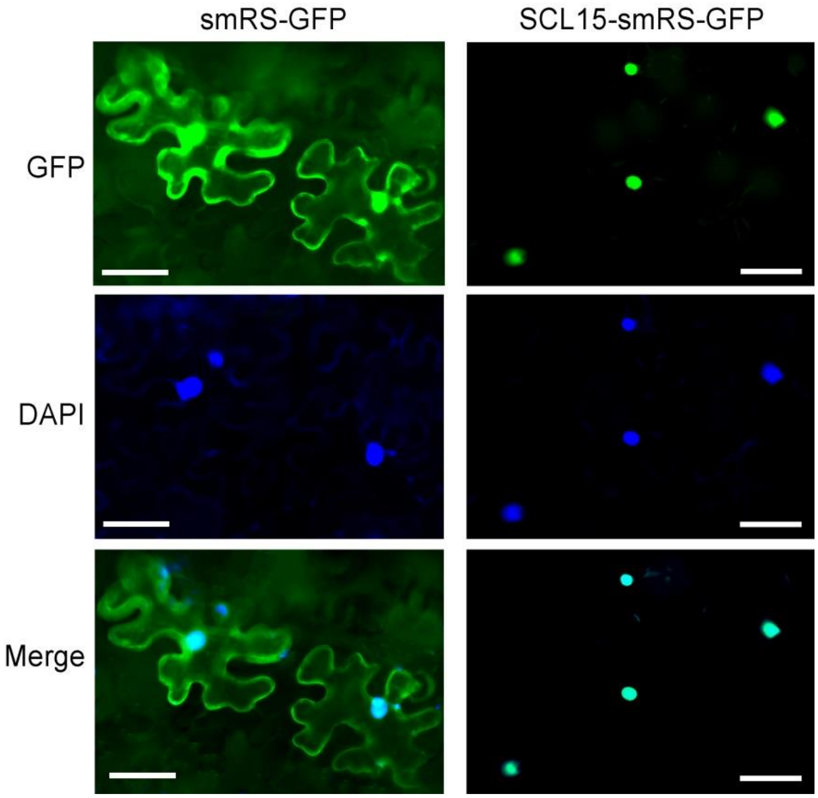

Supplementary Figure 3. Subcellular localization of SCL15. A green GFP fluorescent signal is present in the nuclei of tobacco leaf epidermal cells when a translational fusion between SCL15 and the soluble-modified red-shifted green fluorescent protein (smRS-GFP) (*35Spro::SCL15:smRS-GFP*) was introduced. *35Spro::smRS-GFP* was used as a negative control. Bars=10 µm.

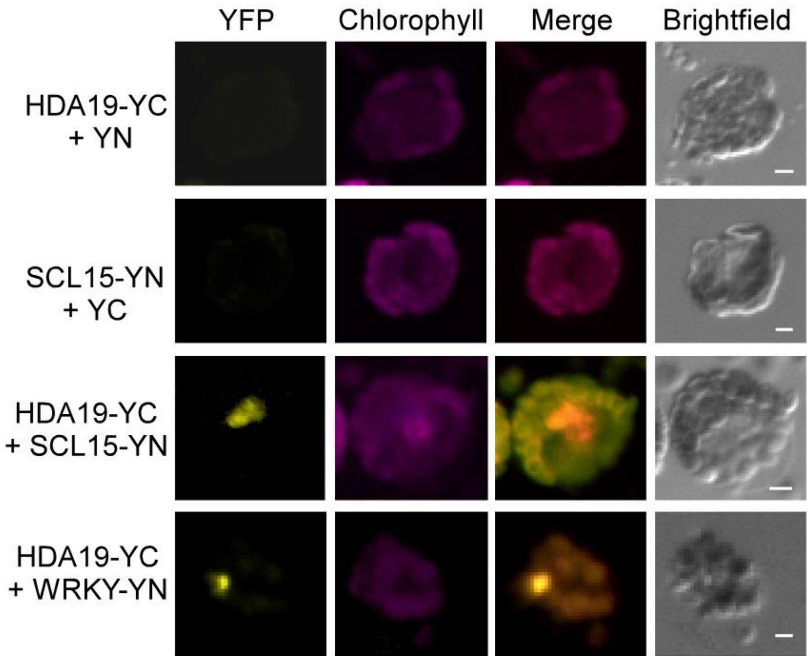

Supplementary Figure 4. BiFC analysis of SCL15 interaction with HDA19 in *Arabidopsis* mesophyll protoplasts. Co-expression of SCL15 fused with the N-terminus of YFP (YN) or HDA19 fused with the C-terminus of YFP (YC) with empty vector YC or YN was used as a negative control and co-expression of HDA19-YC and WRKY38-YN served as a positive control for the BiFC assay. Bars = 5 µm.

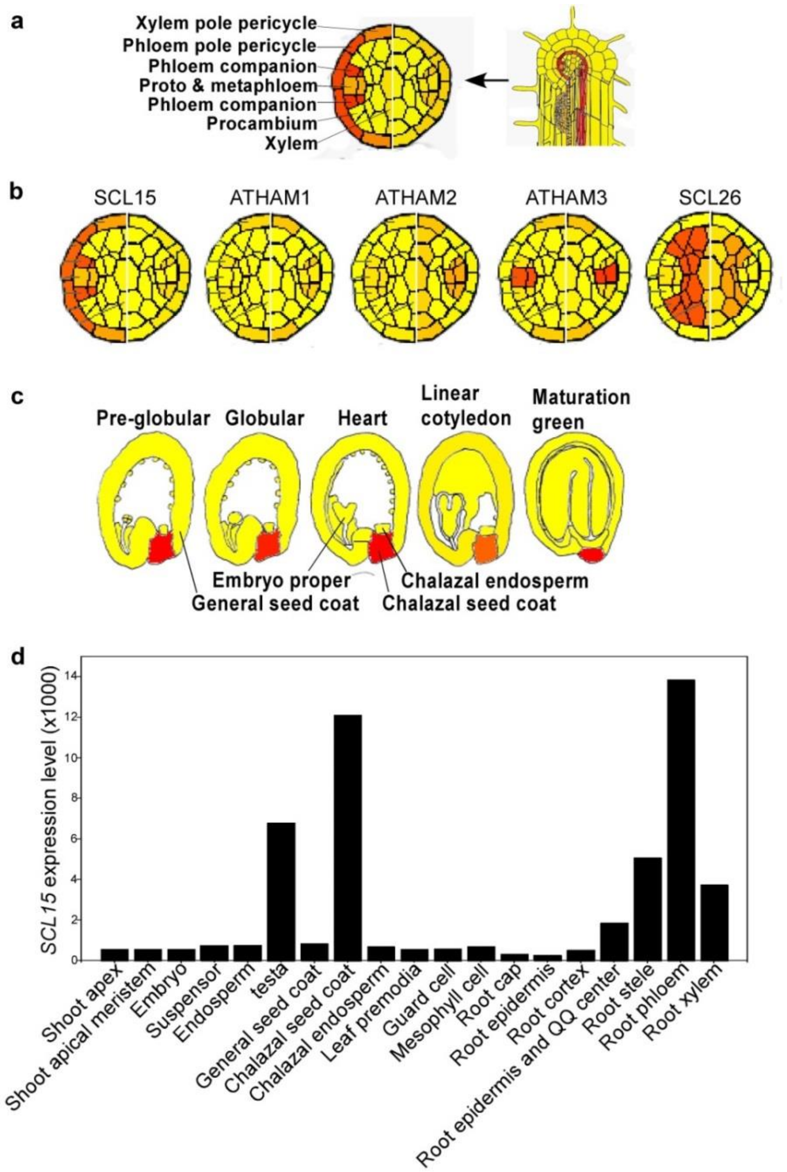

Supplementary Figure 5. Expression patterns of *SCL15* in specific cells and tissue types. (**a**) *SCL15* expression in roots. Note the transcript abundance of *SCL15* is high in the phloem system of vascular tissues, particularly in the phloem companion cells and pericycle cells. (**b**) Comparison of cell type-specific expression patterns in *Arabidopsis* roots between *SCL15* and other members of the ATHAM1 clade as shown in Supplementary Figure 2. (**c**) *SCL15* expression in developing seeds showing marked expression in chalazal seed coat. (**a** to **c**) Data used are retrieved from the BAR *Arabidopsis* eFP browser (http://bar.utoronto.ca/efp/cgi-bin/efpWeb.cgi). (**d**) *SCL15* expression in specific tissues. Data used are derived from Genevestigator (https://www.genevestigator.com/gv/plant.jsp).

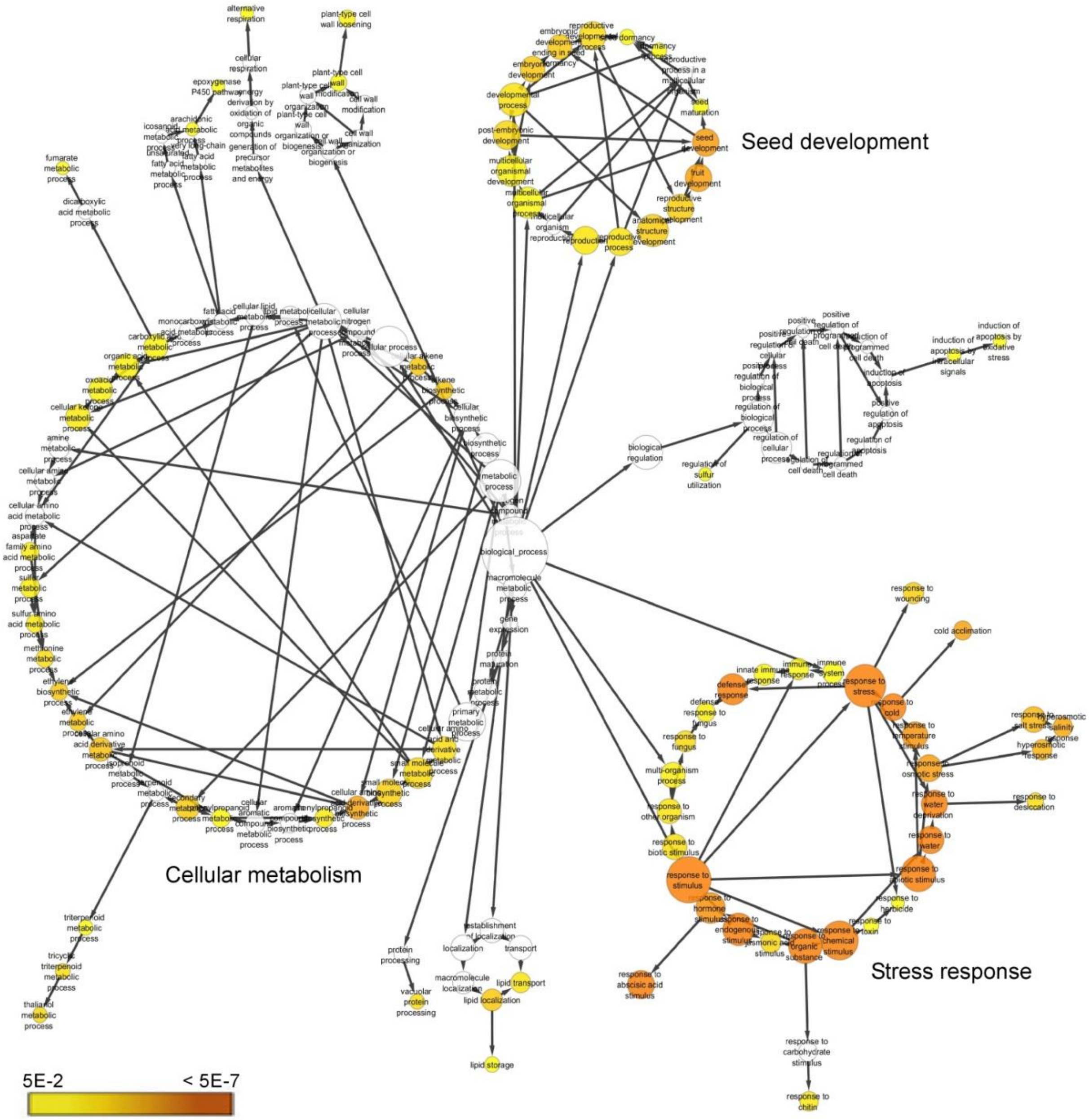

Supplementary Figure 6. Overrepresentation of gene ontology categories among genes up-regulated in *scl15* mutant seedlings. BiNGO analysis representing overrepresented categories of the ontology Biological Process among the genes that are more than three-fold (FDR ≤ 0.05) up-regulated in the *scl15-1* mutant plants compared to wild type (Supplementary dataset 1). The two most overrepresented GO categories are shown. The size of the nodes is proportional to the number of genes annotated to that node. Coloured nodes ranging from yellow to dark orange represent the levels of significance of the overrepresented GO terms with *P* values from 5E-2 to 5E-7. White nodes represent GO terms that are not significantly overrepresented. Statistical testing was as described by Maere et al.[34].

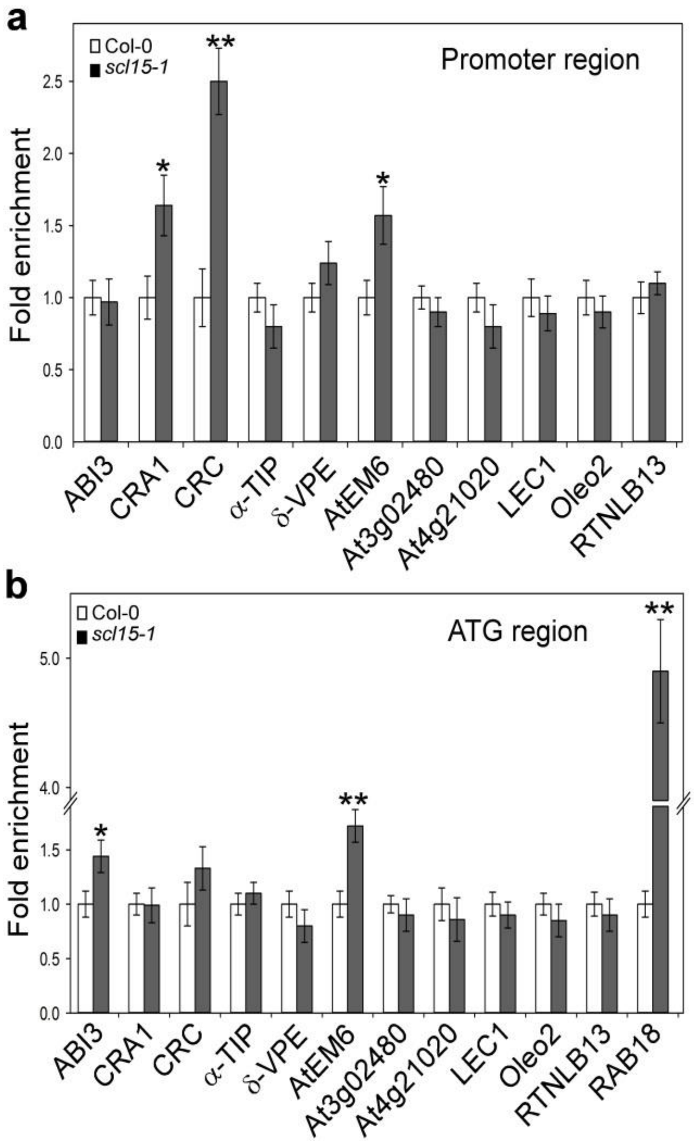

Supplementary Figure 7. ChIP analysis of H4ac levels for the ectopically-expressed seed maturation genes in wild-type Col-0 and the *scl15-1* seedlings. (**a**) Relative H4K5K8K12K16ac levels at the proximal promoter regions. (**b**) Relative H4K5K8K12K16ac levels at the ATG regions. The fold enrichment of *scl15-1* (black bars) relative to the wild type (white bars) is shown and the values are means ± SD of three biological replicates (Student's *t*-test, **P* < 0.05, ***P* < 0.01).

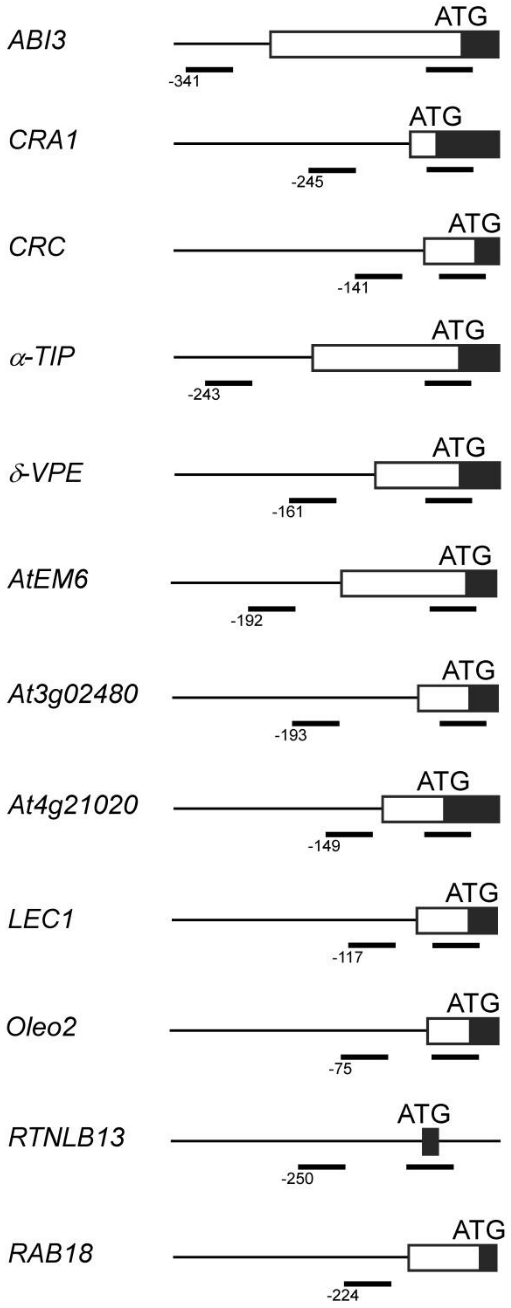

Supplementary Figure 8. Schematic diagram of the regions examined by ChIP. The regions that are located at the promoter and translation start (ATG) regions for the up-regulated embryonic genes in *scl15-1* seedlings are indicated.

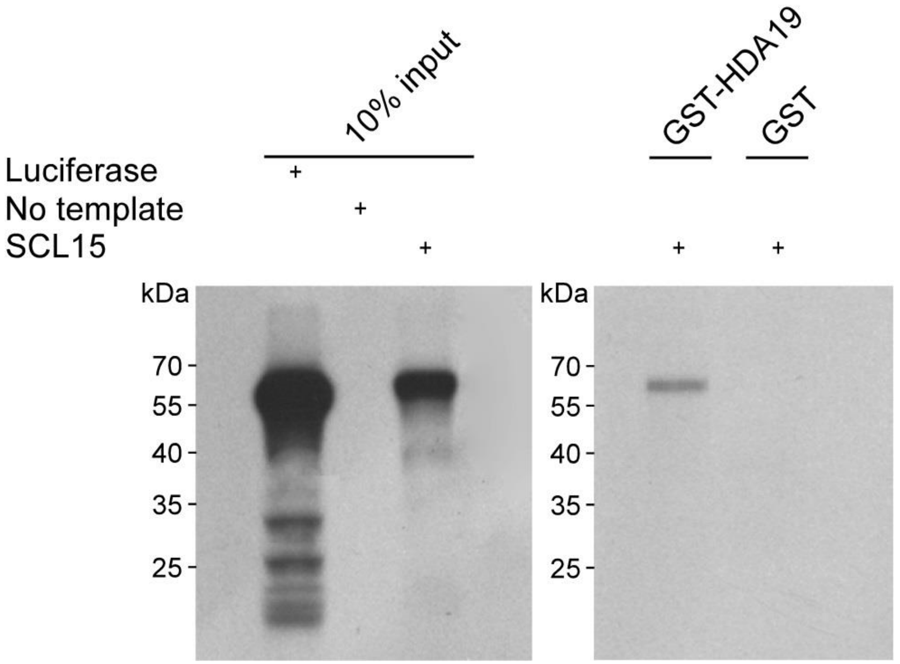

Supplementary Figure 9. GST pull-down assay showing interaction of SCL15 with HDA19 *in vitro*. SCL15 was expressed and radiolabeled using *in vitro* transcription/translation reactions. The translation product (input) was incubated with GST-HDA19 fusion or GST alone.

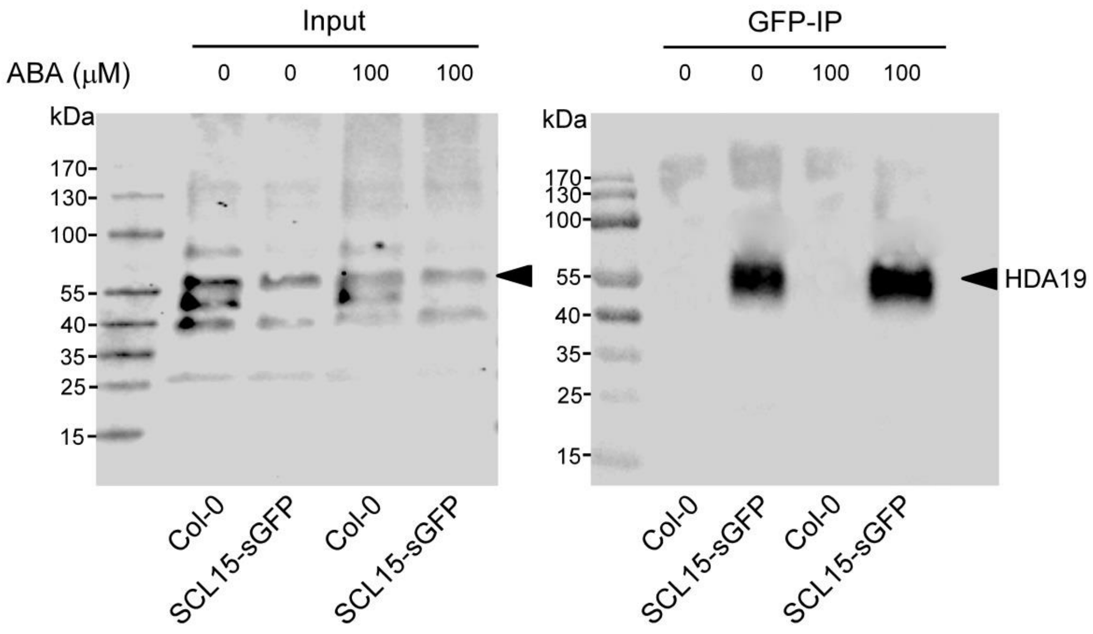

Supplementary Figure 10. Co-IP assay in *Arabidopsis* seedlings showing co-precipitation of HDA19 with SCL15-sGFP. Nuclear extracts (Input) from wild-type Col-0 or homozygous transgenic plants stably expressing *SCL15pro::SCL15-sGFP* treated with or without ABA were immunoprecipitated with a polyclonal anti-GFP antibody (GFP-IP) followed by western blot analysis with polyclonal anti-HDA19 antibodies.

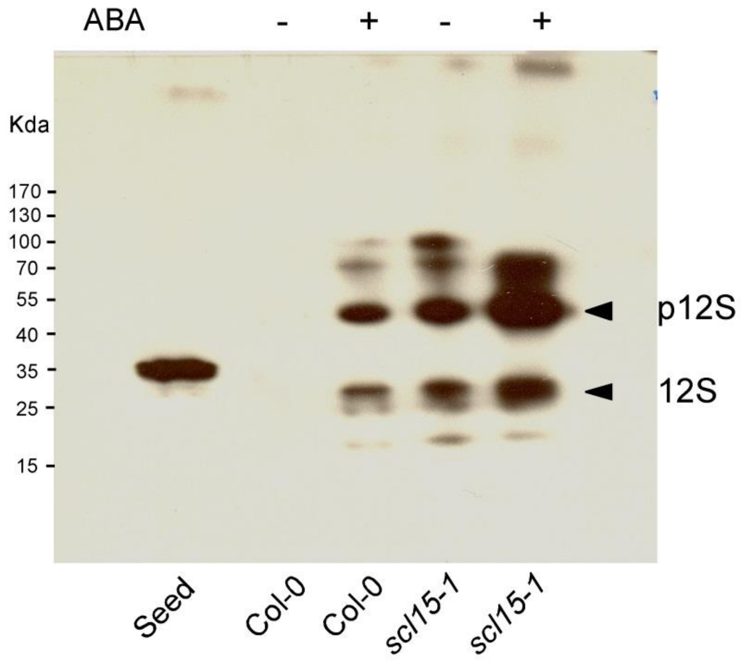

Supplementary Figure 11. Accumulation of 12S globulin in *scl15-1* seedlings.

Immunoblot analysis with monoclonal anti-CRA1 antibody showing that accumulation of proglobulin (p12S) and 12S globulin species in *scl15-1* seedlings is increased by ABA treatment (50 µM) for 2 d.

Supplementary Table 1. Selected seed-related genes repressed by SCL15 in seedlings

Protein	Gene	AGI Code	Fold change
12S globulin	*CRA1*	At5g44120[a]	22.36
	CRC	At4g28520[a]	2.14
Oleosin	*OLEO1-L*	At2g25890[a]	10.36
	OLEO2	At5g40420[a]	4.04
	OLEO3	At5g51210	2.29
Vacuolar processing enzyme	*δ-VPE*	At3g20210[a]	32.51
	β-VPE	At1g62710[a]	3.81
Protein storage vacuole	*α-TIP*	At1g73190[a]	14.28
Cysteine peroxiredoxin	*PER1*	At1g48130[a]	20.03
Cysteine proteinase		At3g54940[a]	7.23
		At4g11320[a]	6.11
		At1g29090	3.75
Reticulon family protein	*RTNLB13*	At2g23640[a]	6.38
Late embryogenesis abundant		At3g17520[a]	20.78
(LEA) protein		At2g35300[a]	15.67
	Em6	At2g40170[a]	9.71
		At3g15670	9.27
		At4g21020[a]	7.09
	M17	At2g41260	6.76
		At3g53040	5.40
		At5g06760	5.27
		At4g36600	5.27
	RAB18	At5g66400[a]	4.82
		At1g52690	3.73
		At1g72100	3.63
		At5g44310	3.48
Cupin family protein		At2g28490	3.47
		At2g18540	2.66
Carbohydrate metabolic enzyme	*BGLU19*	At3g21370	24.46
	UGT85A5	At1g22370	4.52
Peroxiredoxin	*PER1*	At1g48130[a]	20.03
Protein phosphatase 2C (PP2C)		At5g59220	4.99
		At1g07430	2.92
		At5g51760	2.69
Protease inhibitor		At2g38870	3.69
Pseudogene		At5g35660	3.25
Expansin	*EXP2*	At5g05290	3.18
	EXPL1	At3g45970	3.46
ABA-responsive protein		At3g02480[a]	23.45
	ATHVA22B	At5g62490	11.13
	ABI3	At3g24650[a]	2.34
Embryo-specific protein		At1g05510	4.64
	ATS1/CLO1	At4g26740	2.24
Expressed protein		At5g45690	6.09
		At1g16850	4.53
		At1g78070	3.69
		At5g24130	3.57

[a]Gene expression has been validated by qRT-PCR analysis.
